# Expression of Immune Checkpoints in Malignant Tumors: Therapy Targets and Biomarkers for the Gastric Cancer Prognosis

**DOI:** 10.3390/diagnostics11122370

**Published:** 2021-12-16

**Authors:** Danzan Mansorunov, Natalya Apanovich, Pavel Apanovich, Fatimat Kipkeeva, Tatyana Muzaffarova, Anna Kuzevanova, Maxim Nikulin, Olga Malikhova, Alexander Karpukhin

**Affiliations:** 1 Research Centre for Medical Genetics, 1 Moskvorechye St., 115522 Moscow, Russia; gah3ah@mail.ru (D.M.); apanovichn81@mail.ru (N.A.); apanovich2004@mail.ru (P.A.); foty_k@mail.ru (F.K.); tatiana.muzaffarova@mail.ru (T.M.); anka.kuzevanka@yandex.ru (A.K.); 2 N.N. Blokhin National Medical Research Center of Oncology of the Ministry of Health of Russia, 24 Kashirskoe Shosse, 115478 Moscow, Russia; maximpetrovich@mail.ru (M.N.); malikhova@inbox.ru (O.M.)

**Keywords:** immune checkpoint, expression, survival, therapy, target, immune response, biomarkers

## Abstract

To increase the effectiveness of anticancer therapy based on immune checkpoint (IC) inhibition, some ICs are being investigated in addition to those used in clinic. We reviewed data on the relationship between PD-L1, B7-H3, B7-H4, IDO1, Galectin-3 and -9, CEACAM1, CD155, Siglec-15 and ADAM17 expression with cancer development in complex with the results of clinical trials on their inhibition. Increased expression of the most studied ICs—PD-L1, B7-H3, and B7-H4—is associated with poor survival; their inhibition is clinically significant. Expression of IDO1, CD155, and ADAM17 is also associated with poor survival, including gastric cancer (GC). The available data indicate that CD155 and ADAM17 are promising targets for immune therapy. However, the clinical trials of anti-IDO1 antibodies have been unsatisfactory. Expression of Galectin-3 and -9, CEACAM1 and Siglec-15 demonstrates a contradictory relationship with patient survival. The lack of satisfactory results of these IC inhibitor clinical trials additionally indicates the complex nature of their functioning. In conclusion, in many cases it is important to analyze the expression of other participants of the immune response besides target IC. The PD-L1, B7-H3, B7-H4, IDO1 and ADAM17 may be considered as candidates for prognosis markers for GC patient survival.

## 1. Introduction

Recently, therapy based on inhibition of immune checkpoints (ICs) has become the most promising approach in oncology. In practice, antibodies to PD-1/PD-L1 are mainly used as immune checkpoint inhibitors (ICIs). Despite the mostly good results of ICI therapy, a small proportion of patients responding to treatment remain a problem. In this regard, inhibition of an additional IC, such as CTLA-4, is used; other ICIs are being investigated. An increased level of PD-L1 expression in a tumor is generally recognized as the most important predictor of the response to IC inhibition [1,2]. The expression of ICs in a tumor can also be considered as associated with tumor progression, because the immune response and, accordingly, the possibility of tumor development, may depend on its level. Therefore, the level of ICs expression in a tumor can also serve as a prognostic marker. Of particular interest is the question of the possible relationship between the IC expression as a prognostic marker with the possibility of assessing the therapeutic efficacy of the studied IC inhibition. The present work is a based on the literature analysis attempt to answer these questions. Although there are some reviews on ICs and their expression [3,4,5,6], the topic has not been considered in such context.

Gastric cancer (GC) is one of the most common malignancies, ranking 5th in the world in morbidity and 2nd in mortality [7]. A big problem is the late diagnosis of GC and resistance to chemotherapy, as a result of which the 5-year survival rate is around 20% [8]. Thus, there is a need to develop more effective approaches to the GC treatment, one of which may be personalized immunotherapy based on IC inhibition and predicting its effectiveness using IC expression.

This review examines the prognostic value and association of expression with cancer clinical characteristics, as well as the response to the inhibition of a number of ICs expressed in solid tumor: B7-H1 (PD-L1, CD274), B7-H3 (CD276), B7-H4 (VTCN1), Galectin-3, Galectin-9, IDO1, CEACAM1 (CD66a), CD155 (PVR), Siglec-15 and cell surface protease ADAM17 (CD156b, TACE), which, although not an IC, is important in modulating the immune response and progression of tumors.

## 2. Immune Checkpoint Expression in Connection with Clinical Features and the Therapeutic Efficacy of Their Inhibition

**Figure 1:**
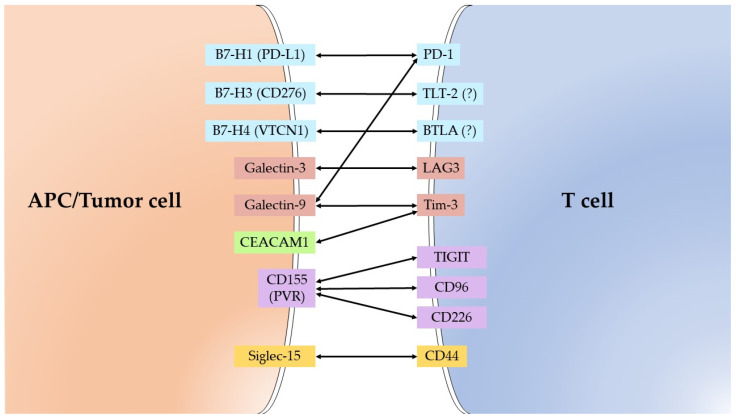
The ligands of immune checkpoint (left) and their receptors. (?) – putative receptor.

The analyzed IC ligands, expressed in tumors, and their receptors on T-cells are presented on Figure 1 and, in more details, in Table 1.

### 2.1. PD-L1 (B7-H1)

Programmed death 1 (PD-1) is a checkpoint protein and a member of the CD28 family. PD-1 is an important immunosuppressive molecule whose biological function is to maintain the T-cell response within the physiological range. PD-1 regulates T-cell activation by interacting with its ligands, programmed death ligand-1 (PD-L1, B7-H1, CD274) and programmed cell death 1 ligand 2 (PD-L2, B7-DC, CD273). The PD-1 is encoded by *PDCD1* gene with five exons, while the PD-L1 is encoded by *CD274* gene with seven exons [3]. Interaction of PD-1 with its ligands leads to an inhibitory effect on the activation of T-lymphocytes, an increase in the number of Foxp3+ T-regulatory cells and inducing of apoptosis of CD8+ cytotoxic T-lymphocytes, which allows the tumor to “escape” the immune response [9]. This interaction inhibits CD4+/CD8+ tumor-infiltrating T-lymphocytes (CD4+/CD8+ TILs), which results in a decrease in cytokines, such as Interleucin-2 (IL-2), interferon gamma (IFN-γ) and tumor necrosis factor (TNF). PD-L1 on the surface of tumor cells can be enhanced by IFN-γ produced by activated T-cells [10]. PD-L1 expression is upregulated through signaling pathways, including PI3K/Akt/mTOR and PD-L1-mediated immunoresistance could be suppressed by PI3K kinase pathway inhibitors. ERK/P38 MAPK pathway in tumor cells may be upregulated by overexpression of PD-L1. Activation of the Hedgehog pathway in GC leads to increased PD-L1 expression and immunotherapy resistance. Improvement of treatment in GC patients through combination drug therapy with inhibition of ICs and Hedgehog pathway is proposed as possible [3]. PD-L1 is expressed on the surface of T-, B-lymphocytes, macrophages, dendritic cells (DCs), and bone marrow mast cells [11]. Expression of PD-L1 in tumor cells is observed in many types of malignant tumors, such as melanoma, kidney, lung, ovarian, colon, stomach, esophagus, breast, bladder, cervix, head and neck, liver, and others [2,12]. Thus, in GC and gastro-esophageal junction cancer, PD-L1 is expressed in about 30% of cases, but is not expressed in normal gastric tissue [13].

Expression of PD-L1 as a prognostic marker is widely studied; in most studies, PD-L1 expression is considered as a marker of poor prognosis in various types of malignant tumors. Wu et al. was found that PD-L1 overexpression is associated with the worst 3-year (OR = 2.43, 95% CI = 1.60–3.70, *p* < 0.0001) and 5-year (OR = 2.23, 95% CI = 1.40–3.55, *p* = 0.0008) overall survival (OS) in many types of solid tumors [14]. Similarly, a meta-analysis by Xiang et al. showed that 1-year (RR = 2.02, 95% CI = 1.56–2.60, *p* = 0.039), 3-year (RR = 1.57, 95% CI = 1.34–1.83, *p* < 0.001) and 5-year (RR = 1.43, 95% CI = 1.24–1.64, *p* < 0.001) OS of patients with solid tumors expressing PD-L1 is significantly lower compared to patients with PD-L1-negative tumors [15].

In malignant tumors of the digestive system, PD-L1 expression was a marker of poor prognosis (HR = 1.44, 95% CI = 1.18–1.76, *p* < 0.001), including GC (HR = 1.43, 95% CI = 1.05–1.94, *p* = 0.021) and pancreatic cancer (HR = 2.64, 95% CI = 1.78–3.93, *p* < 0.001) [16].

Several meta-analyses of the PD-L1 expression predictive value in GC have shown that PD-L1 expression is associated with a poor survival rate [17,18,19,20,21].

At the same time, it should be noted that there are some data on a good prognosis of PD-L1 expression in GC [13,22], as well as the absence of a relationship between PD-L1 expression and prognosis [23]. The reasons for these contradictory results have to be clarified in the future. However, it should be noted that in most studies, PD-L1 expression level is measured by IHC, using reagents from different manufacturers, with different cut-offs, as well as on samples of patients from different countries.

Measurement of PD-L1 expression level in malignant tumors can be carried out not only in the tumor material, but also in the blood in order to determine its soluble form (sPD-L1). Thus, in a meta-analysis by Huang et al., high serum sPD-L1 level measured by ELISA was associated with poor OS (HR = 1.85, 95% CI = 1.59–2.15) in 17 tumor types, both solid and non-solid [24]. In GC, high serum sPD-L1 level was a prognostic factor of poor OS [25]. In addition to blood serum, PD-L1 expression level is measured in peripheral blood cells by qRT-PCR or flow cytometry. In a study from Ito et al., high PD-L1 mRNA was an independent prognostic factor of poor OS [26].

Some meta-analyses have revealed an association of PD-L1 expression not only with OS, but also with other clinical characteristics. For example, in a meta-analysis from Gu et al., an association of PD-L1 expression in GC with such clinical characteristics as the depth of tumor invasion (*p* = 0.03), lymph node metastasis (*p* = 0.03), venous invasion (*p* = 0.0003), Epstein-Barr virus (EBV) infection (*p* < 0.0001), microsatellite instability (MSI) (*p* = 0.0001) was found [18]. In another meta-analysis, PD-L1 expression in GC was associated with lymph node metastasis (OR = 1.73, 95% CI = 1.18–2.54, *p* < 0.001) [17]. In the study of Zhang et al. there was an association of PD-L1 expression with tumor size and lymph node metastasis [19]. The clinical characteristics associated with increased expression of PD-L1 also indicate a poor prognosis for the development of GC.

PD-L1 expression is also associated with a better prognosis for therapy by PD-1 inhibitor. A meta-analysis of publications on the use of PD-1 inhibitors in metastatic GC showed that ICIs significantly improved OS in patients with PD-L1-positive tumors [27].

The complex of considered results demonstrates the correlation of poor prognosis due to increased expression of PD-L1 and the effectiveness of ICI therapy, which blocks its interaction with the receptor.

### 2.2. B7-H3 (CD276)

B7-H3 (CD276) is a transmembrane glycoprotein, a member of the B7 immunoregulatory family. The role of CD276 in the regulation of the immune response initially looked controversial. CD276 was originally described as a co-stimulatory molecule involved in the proliferation of both CD4+ and CD8+ T-cells, enhancing the induction of cytotoxic T-cells [28]. The *CD276* gene is located on chromosome 15. Extracellular domain of CD276 contains two similar pairs of the immunoglobulin V (IgV) and IgC domains due to exon duplication [3]. Hashiguchi et al. reported that binding of CD276 to its receptor TLT-2 increased T-cell proliferation, cytokine production and cytotoxicity, and the use of antibodies against CD276 and TLT-2 significantly suppressed T-cell activation [29].

At the same time, more data are emerging on the inhibitory properties of CD276. The proliferation of both CD4+ and CD8+ T-cells could be inhibited by CD276 [30]. CD276 inhibited the proliferation of both CD4+ and CD8+ T-cells and reduced the production of IL-2 and IFN-γ, possibly through suppression of NF-κB-, NFAT-, AP-1-mediated signaling pathways. Blocking CD276 binding with antagonistic antibodies significantly increased T-cell proliferation and IL-2 levels [30,31].

Decreased expression of CD276 results in decreased expression of proteins associated with metastasis, such as MMP-2, STAT3, and IL-8 [32]. It has also been shown that CD276 expression promotes migration and invasion of tumor cells [33,34].

In GC with a high expression of CD276, a reduced density of CD8+ T-cells was observed in the center of the tumor, which suggests the involvement of CD276 in the mechanisms of tumor escape from the immune response [35].

Li et al. reported that CD276 could increase the radioresistance of GC cells by modulating apoptosis, cell cycle progression, and double-stranded DNA breaks. It was found that overexpression of CD276 suppresses autophagy in gastric tissue and cells [36].

Increased expression of CD276 has been observed in activated B-cells, T-cells, NK-cells, malignant tumors of the colon, ovary, prostate, pancreas, kidney, squamous cell carcinoma, non-small cell lung cancer, and GC, but is not expressed, or is expressed at a low level, in most normal cells [37,38]. This makes it possible to consider CD276 as a promising target for immunotherapy.

There is a lot of evidence of the association of CD276 expression with poor prognosis. In two meta-analyses, increased expression of CD276 in many types of malignant tumors was associated with poor OS (HR = 2.09, 95% CI = 1.60–2.74, *p* < 0.001; HR = 1.58, 95% CI = 1.32–1.90, *p* < 0.00001, respectively) [37,39]. Increased expression of CD276 in GC is usually associated with poor survival [40,41].

The results of meta-analyses indicate a relationship between increased expression of CD276 with poor survival in patients with GC and other types of malignant tumors. However, there are also some data on better OS with increased expression of CD276 in patients with GC [42] and on the absence of association of expression with prognosis [35].

In GC, the expression of CD276 is also associated with other clinical characteristics. Zhan et al. reported that CD276 expression significantly correlated with stage, depth of tumor infiltration, and lymph node involvement [40]. In Li et al., the level of CD276 expression was positively correlated with the depth of tumor infiltration (*p* = 0.005) [34].

That is, clinical characteristics with increased CD276 expression also indicate a poor prognosis in patients with GC. An anti-CD276 monoclonal antibody (mAb) was developed for cancer therapy [43], which, under the name Enoblituzumab, is undergoing phase 1–2 clinical trials [44,45,46], that demonstrate the antitumor activity of the drug and an acceptable safety profile [47,48].

Consequently, in the process of research, the understanding of the relationship of increased expression of CD276 with a poor prognosis in different types of tumors, including GC, was achieved and the prospects of its use as a target for ICI have been strengthened.

### 2.3. B7-H4 (VTCN1)

B7 homolog 4 (B7-H4), also known as VTCN1, is a co-inhibitory IC ligand from the B7 family that negatively modulates CD4+ and CD8+ T-cell activity and innate immunity [49]. B7-H4 is a type I transmembrane protein containing 282 amino acid residues, which has been classified as containing an amino-terminal extracellular domain, a large hydrophobic transmembrane domain and an intracellular domain with only 2 amino acids. Like other members of the B7 family, B7-H4 has a pair of Ig-like regions in the extracellular domain [50]. The *B7-H4* gene is mapped on chromosome 1p13.1, consists of six exons, of which exon 6 is involved in alternative splicing with the generation of two different transcripts. Human pseudogene B7-H4 is located on chromosome 20p11.1 [51]. The B7-H4 receptor is currently not proven; although it is indirectly assumed to be a B and T lymphocyte attenuator (BTLA) [52]. The interaction of B7-H4 and its receptor on T-cells leads to a decrease in the proliferation of both CD4+ and CD8+ T-cells [53].

B7-H4 mRNA is widely expressed in a variety of tissues, but in normal tissue protein expression is limited, suggesting post-transcriptional regulation [54]. Expression of B7-H4 may be induced on T-cells, B-cells, monocytes, and DCs after stimulation with LPS, PHA, IFN-γ, PMA, or ionomycin [55].

B7-H4 is overexpressed in a wide range of malignancies, including cancers of the stomach, kidneys, ovaries, lungs, uterus, breast, prostate, and skin [56]. B7-H4 overexpression activates NF-κB signaling and upregulates the epithelial–mesenchymal transition (EMT) inducers Twist1 and Snail; in addition, it promotes EMT by decreased E-cadherin and increased vimentin expression [57]. B7-H4 expression on the nuclear membrane is significantly associated with the Ki-67 index, which suggests a greater proliferative potential of tumors with B7-H4 expression [58].

B7-H4 significantly reduces the number of T-cells in the S phase and increases the number of cells in the G0/G1 phase, as well as arrests cell cycle progression of T-cells in the G0/G1 phase [55].

B7-H4+ neutrophils positively correlated with increased GM-CSF detection. GC tumor-derived GM-CSF activated neutrophils and induced neutrophil B7-H4 expression via JAK/STAT3 signaling pathway activation. Additionally, higher intratumoral B7-H4+ neutrophil percentage/number was found in GC patients with advanced TNM stage and reduced OS following surgery [59].

B7-H4 expression in many types of solid tumors is associated with OS and clinical and pathological characteristics, including high tumor stage and grade, lymph node metastasis, early recurrence, and negatively correlated with the density of tumor-infiltrating T-lymphocytes [54].

In a meta-analysis by Song et al., it was shown that increased B7-H4 expression is associated with worse OS in solid tumors (HR = 1.79, 95% CI = 1.56–2.06, *p* < 0.001) [60]. A meta-analysis by Cui et al. also showed an association of increased B7-H4 expression with a poor GC prognosis [61]. Expression of B7-H4 in GC was measured in tumor tissue both by IHC and RT-PCR [62,63,64,65], in peripheral blood by RT-PCR [66], and in serum by ELISA [67].

The association of B7-H4 expression with such clinical and pathological characteristics as the TNM stage, the depth of invasion, and metastasis to the lymph nodes was also reported [62,63,64,66,67]. In addition, there was a positive correlation between B7-H4 expression and the number of Foxp3+ Tregs in the tumor [64], but an inverse correlation with the number of TILs [63].

Consequently, among patients with GC and other solid tumors increased B7-H4 expression in tissue, blood, and serum, studied by different methods, including IHC, RT-PCR, and ELISA, was associated with a poor prognosis. Inhibition of B7-H4 by mAb FPA150 in clinical study NCT03514121 demonstrated acceptable safety despite B7-H4 expression in many tissues, and some therapeutic effect among B7-H4 expressing tumors [68], indicating that this IC is promising as a therapeutic target.

### 2.4. Galectin-3

Galectin-3 (Gal-3) is a member of the galectin family that is widespread in mammalian tissues and is determined by its carbohydrate recognition domains (CRDs) with a specific binding affinity for β-galactosides. Gal-3 is the only chimeric type galectin; it contains one CRD that is connected to an extended non-lectin N-terminal domain [69]. In humans, Gal-3 is a 35 kDa protein encoded by the *LGALS3* gene located on chromosome 14 [70]. Gal-3 is expressed in both intracellular and extracellular spaces, such as the cell surface or extracellular matrix (ECM), and its localization depends on tissue, cell type, cell proliferative state, and differentiation level [71].

The different location of the Gal-3 contributes to its different functions. In the cytoplasm, Gal-3 is important for cell survival due to its interactions with certain proteins associated with survival, including B-cell lymphoma-2 (Bcl-2) and activated guanosine-5’-triphosphate (GTP)-bound K-Ras. In the nucleus, Gal-3 promotes pre-mRNA splicing and regulates gene transcription, while extracellular Gal-3 modulates intercellular interactions, including between epithelial cells and the ECM. Gal-3 is widely expressed in human tissues and in all types of immune cells (macrophages, monocytes, DCs, eosinophils, mast cells, NK-cells, and activated T- and B-cells), epithelial cells, endothelial cells, and sensory neurons [72]. Altered expression and localization of Gal-3 are involved in the regulation of cancer cell growth, transformation, apoptosis, immunosuppression, angiogenesis, adhesion, invasion, and metastasis [73]. Decreased Gal-3 expression leads to a decrease in adhesion between tumor cells and facilitates the invasion of cancer cells [74].

One of the Gal-3 receptors is LAG-3 (CD223), an inhibitory receptor that negatively regulates proliferation, activation, and homeostasis of both CD8+ and CD4+ T-cells. Gal-3 affects the antitumor immune response by suppressing activated antigen-committed CD8+ T-cells through the expression of LAG-3 in the tumor microenvironment (TME) and inhibiting the growth of plasmacytoid DCs [75].

Extracellular Gal-3 regulates various T-cell functions, including TCR signaling, IL-5 production, migration, adhesion, and apoptosis. Extracellular Gal-3 induces T-cell apoptosis, while intracellular Gal-3 inhibits it [76]. Pentameric Gal-3 forms lattices with cell surface glycans, such as TCR, EGFR and TGF-β [69]. Complex lattices have been identified that assemble between galectins and various receptors to facilitate T-cell death or inhibit its function. Additionally, it was shown that Gal-3 sequesters IFN-γ in the ECM of the tumor, decreasing the infiltration of T-cells [77].

In the nucleus, Gal-3 plays a key role in the regulation of expression of associated with tumor progression genes, such as cyclin D1, TTF-1 and MUC2 [78]. Gal-3 overexpression enhances the activation of K-Ras, the most important Ras oncoprotein in human tumors [79].

The expression of Gal-3 differs depending on the type of malignant tumor. For example, in basal cell and squamous cell carcinomas, reduced expression of Gal-3, while in melanoma, increased expression was observed [80]. There is evidence of an association of Gal-3 expression with prognosis. Thus, in the Wang et al. meta-analysis, increased Gal-3 expression was associated with decreased OS or DFS/RFS/PFS in many types of solid tumors [81]. The association of increased Gal-3 expression with a poor prognosis was shown by meta-analyses for malignant tumors of the digestive system, such as colorectal cancer [82] and hepatic cancer [83].

However, in GC, the opposite situation was observed: in the meta-analysis by Long et al., decreased Gal-3 expression was associated with a poor prognosis [84]. The association of Gal-3 expression with the other clinical characteristics of GC is also being investigated. Okada et al. reported that reduced Gal-3 expression was correlated with lymph node metastasis, lymphatic invasion and TNM stage [71]. Moreover, increased Gal-3 expression was related to less advanced depth of invasion, the absence of lymph node metastasis, and distant metastasis, low TNM stage and the absence of lymphovascular invasion [85]. In another work, the opposite result was obtained: GC with lymph node metastasis showed an increased Gal-3 mRNA expression level in tumor tissues compared to those without lymph node metastasis [86]. There was a significant correlation between the degree of Gal-3 expression and tumor progression as classified by TNM staging [87]. In addition, high Gal-3 serum level was associated with lymph node metastasis and distant metastasis [88].

As can be seen from the results of cited studies, the data on the relationship of Gal-3 expression with clinical characteristics, including patient survival, are significantly contradictory. It can be assumed that this phenomenon is conditioned by the strong dependence of the research result on the set of specific characteristics of the samples due to the complex action of Gal-3 described above. Apparently, these properties are due to the failure of several clinical studies using an anti-Gal-3 antibody (GM-CT-01) for antitumor therapy [89].

### 2.5. Galectin-9

Galectin-9 (Gal-9) is also a member of the galectin family. Gal-9 belongs to tandem-repeat-type galectins and consists of two different CRDs in one polypeptide chain, separated by a linker sequence [69]. In human, Gal-9 is encoded by the *LGALS9* gene located on the long arm of chromosome 17 at locus 11.2 (17q11.2) [90].

Gal-9 activates signaling cascades necessary for the stimulation of innate immunity, promotes the maturation of DCs and attracts neutrophils and eosinophils to the infection site. Gal-9 interacts with cell surface receptors to elicit transient signals leading to the production of pro-inflammatory chemokines and cytokines by activated T-cells. Intra- and extracellular changes in concentration of Gal-9, receptors available for binding and distortion of neighboring cells signals lead to physiological changes.

Gal-9 is a ligand for Tim-3, an exhaustion marker expressed by activated T-cells. The interaction of Gal-9 with Tim-3 induces apoptosis of peripheral T-cells ex vivo via the calcium-calpain-caspase-1 pathway [91]. Moreover, most Tim-3+ T-cells of the tumor co-express PD-1, whose ligand may also be Gal-9. By binding to Gal-9, PD-1 promotes the retention of PD-1+ Tim-3+ T-cells and attenuates Gal-9/Tim-3-induced cell death [92].

Gal-9 is widely expressed in tissues of the immune system, such as the spleen and thymus, in several types of immune cells, including T-cells, B-cells, macrophages and mast cells, as well as in tissues of endodermal origin, such as liver, intestine, stomach and lungs [90,93]. Gal-9 expression is significantly altered in most malignant tumors [92], Gal-9 is overexpressed in GC tumor cells [94].

In recent years, more and more attention has been paid to the prognostic value of Gal-9 expression in various types of tumors [95,96,97].

A meta-analysis by Zhou et al. investigating the predictive value of Gal-9 expression in solid tumors found improved OS with increased Gal-9 expression (HR = 0.70, 95% CI = 0.51–0.71, *p* = 0.006) [98]. In another meta-analysis, increased Gal-9 expression was also an indicator of better tumor-specific survival in solid tumors (HR = 0.48, 95% CI = 0.39–0.58, *p* < 0.001) [99]. The predictive value of increased Gal-9 expression in GC, on some data, is likely associated with a good prognosis. In Jiang et al. and Choi et al., increased Gal-9 expression was associated with better OS [100,101]. However, Wang et al. reported that OS was poor (*p* = 0.0028) in patients with increased expression of Gal-9 in the tumor [94]. A similar result was obtained when analyzing the data presented in The Cancer Genome Atlas (TCGA) and the Cancer Cell Line Encyclopedia (CCLE) in many tumor types, including GC [92].

An association of Gal-9 expression with different clinical characteristics has been investigated. Gal-9 suppresses metastasis by inhibiting several steps required for tumor metastasis: detachment from tumors, invasion of the ECM, and attachment to the vascular endothelium [101]. In solid tumors, increased expression of Gal-9 significantly correlated with a lower depth of invasion (OR = 2.80, 95% CI = 1.97–3.96, *p* < 0.001), an earlier histopathological stage (OR = 3.00, 95% CI = 2.04–4.42, *p* < 0.001), the absence of lymph node metastasis (OR = 0.47, 95% CI = 0.25–0.89, *p* = 0.020) and the absence of distant metastasis (OR = 13.85, 95% CI = 3.50–54.76, *p* < 0.001) [98]. In GC, decreased expression of Gal-9 was associated with a greater depth of invasion, lymph node metastasis, distant metastasis, and poor TNM stage [100,101].

As we can see, the results for Gal-9 are also far from unambiguous. In this regard, the complex consequences of changes in its expression should be noted. The interaction of Gal-9 with its receptor Tim-3 induces T-cell apoptosis. At the same time, Gal-9 also interacts with PD-1, which inhibits apoptosis. The resulting effect may depend on the ratio of these three components. In addition, inhibition of Gal-9 leads to the accumulation of Treg cells in the TME, which reduces the antitumor immune response [92]. Such a complex system of interactions and their consequences requires, apparently, a deeper characterization of the studied samples and clinical cases in order to understand the discussed relationships. The quantitative level of Gal-9 expression should also be accounted. Schulz et al. obtained opposite results on the association of moderate and high Gal-9 expression with survival [102].

### 2.6. IDO1

Indoleamine-2,3-dioxygenase (IDO1) and tryptophan-2,3-dioxygenase (TDO) are the main enzymes in the first step of the kynurenine pathway, which catabolizes tryptophan to kynurenine. It is known that IDO1 catabolizes most of the tryptophan in various organs, while TDO is mainly expressed in the liver [103]. IDO1 is a monomeric heme-containing protein encoded by *IDO1* gene located on chromosome 8p12. TDO protein is encoded by *TDO2* gene mapped on chromosome 4 and forms a tetrameric heme-containing complex with lower than IDO1 enzyme activity for tryptophan [104]. IDO1 is an inducible enzyme, the most important inducer of which is the cytokine IFN-γ. Hyperactivation of the kynurenine pathway leads to a decrease in tryptophan level and accumulation of kynurenine, which has a toxic effect on T-cells. This can lead to cell cycle arrest of immune cells such as CD8+ T-lymphocytes, NK-cells, and invariant NKT cells through the GCN and mTOR signaling pathways [105]. Inflammation activates the kynurenine pathway and increases the expression of IDO1. Inflammatory cytokines, such as IL-1β, TNF-α and, in particular, IFN-γ stimulate JAK/STAT-mediated expression of IDO1 [106]. Increased expression of IDO1 correlates with a decreased level of infiltrating CD3+ T-cells, CD8+ T-cells, CD57+ NK-cells, B-cells, and increased level of Foxp3+ Treg in various types of malignant tumors [107].

IDO1 and TDO expression was found in various cells in the TME (including metastatic sites) and tumor-draining lymph nodes, including tumor, stromal, vascular, and immune cells [106]. IDO1 is overexpressed in the vast majority of malignant tumors [108]. High IDO1 expression correlates with poor prognosis in many types of cancer [109]. For example, in the meta-analysis by Yu et al. overexpressed IDO1 was associated with poor OS in patients with many types of solid tumors (HR = 2.03, 95% CI = 1.56–2.63). Moreover, patients with high IDO1 expression had poor disease-free survival (HR = 2.47, 95% CI = 1.46–4.20) [105]. Another meta-analysis showed a similar result: increased IDO1 expression was significantly associated with reduced OS in many types of cancer (HR 1.92, 95% CI = 1.52–2.43, *p* < 0.001) [110].

In GC, increased expression of IDO1 is usually associated with poor OS [111,112,113,114]. It should be noted that, in some studies, association of IDO1 expression with survival was not obtained [115,116].

There is evidence of an association of increased IDO1 expression with clinical characteristics of solid tumors, such as tumor differentiation (OR = 1.81, 95% CI = 1.05–3.12, *p* = 0.033), the presence of distant metastasis (OR = 1.45, 95% CI = 1.02–2.06, 0.039), and TNM stage (OR = 1.89, 95% CI = 1.13–3.17, *p* = 0.015) [105]. In GC, IDO1 expression was significantly associated with the depth of tumor invasion and lymph node metastasis [111,117,118].

Thus, increased expression of IDO1 in many types of tumors, including GC, is associated with worse OS and clinical characteristics indicating a poor prognosis. Therefore, unexpected were the unsatisfactory results of numerous clinical studies of anti-IDO1 drugs as mono therapy [108]. These results lead to the conclusion that there is still insufficient understanding of the role of IDO1 in immune suppression and need for further research in this direction [103,108,119]. 

In addition, the therapeutic outcome may be influenced by the predominant functioning of individual immune pathways, which should be taken into account when planning clinical trials [120]. There may be mechanisms that are not directly related to IDO1, which compensate for the effect of blocking it [121]. However, the considered circumstances do not eliminate the possibility of using IDO1 expression as a prognostic marker.

### 2.7. CEACAM1

Carcinoembryonic antigen-related cell adhesion molecule 1 (CEACAM1), also known as cluster of differentiation 66a (CD66a), is a member of the carcinoembryonic antigen cell adhesion molecule (CEACAM) family, which belongs to the Ig superfamily. CEACAM1 is a type I transmembrane protein containing an extracellular N-terminal variable domain followed by up to three constant C2-like Ig domains [122]. The extracellular domains of CEACAM1 are required for homophilic interaction with CEACAM1 and heterophilic interaction with CEA (also known as CEACAM5) as well as T cell-immunoglobulin and mucin-domain containing 3 (Tim-3).

CEACAM1 has 12 isoforms due to alternative splicing of the *CEACAM1* gene. Inclusion or exclusion of exon 7 of the *CEACAM1* gene leads to the splicing of mRNA into transcripts encoding two different cytoplasmic domains. Long isoform has ITIM motifs, while short isoform lacks them. Moreover, CEACAM1 can occur as secreted variants [123].

CEACAM1 is the most common member of the CEACAM family and is expressed in epithelial and endothelial cells, CD4+ and CD8+ T-lymphocytes, DCs, NK-cells, and bone marrow cells [124]. CEACAM1 is not observed in naive T-cells, but is known to be the only member of the CEACAM family expressed by activated T-cells [123].

Tim-3 is the ligand of CEACAM1, which is also a member of the Ig superfamily and expressed as a type I membrane protein. Tim-3 is co-expressed and forms a heterodimer with CEACAM1. The presence of CEACAM1 confers Tim-3 inhibitory function. CEACAM1 promotes the maturation and expression of Tim-3 on the cell surface through the formation of heterodimeric interactions [125].

CEACAM1 expression in T-cells, NK-cells, B-cells, monocytes and granulocytes is important for the progression and development of various types of cancer [123]. Homophilic interactions of CEACAM1 and heterophilic interactions of CEACAM1 with CEACAM5 inhibit NK-mediated destruction regardless of recognition of the MHC class I, and also inhibit the activity of NK-cells and TILs to release IFN-γ [126]. It has been shown that CEACAM1 is the main effector of vascular endothelial growth factor (VEGF) in the formation of tumor microvessels at an early stage [127]. CEACAM1 appears to play an important role in tumor growth and progression by participating in immune evasion mechanisms, as well as an angiogenic effect.

CEACAM1 is expressed in many cancers and correlates with tumor progression, metastasis, and OS [128,129,130]. In GC, the effect of CEACAM1 expression on prognosis is ambiguous, since both increased CEACAM1 expression [131] and loss of CEACAM1 expression [132] were associated with poor prognosis. Increased CEACAM1 expression in GC is associated with such clinical characteristics as TNM stage and lymph node metastasis [133,134]. At present, there are not enough studies on the association of CEACAM1 expression with survival in GC; however, increased CEACAM1 expression is most likely associated with the TNM stage and lymph node metastasis.

There are still insufficient data to conclude on the therapeutic efficacy of CEACAM1 inhibition. The anti-CEACAM1 mAb CM-24 in clinical study NCT02346955 did not show therapeutic efficacy. Another anti-CEACAM1 mAb, PMG1124, showed good results in a mouse model of a human tumor [135,136].

It should be noted that in model systems, inhibition of CEACAM1 by CM-24 also led to good results. Therefore, the results of clinical trials should be waited.

### 2.8. CD155

CD155, also known as poliovirus receptor (PVR) or Necl-5, is an adhesion molecule of the Ig superfamily involved in various physiological processes such as proliferation, migration, cell adhesion and modulation of the immune response [137]. CD155 binds with few cellular receptors and the ECM protein vitronectin. Moreover, CD155 makes a significant contribution to the establishment of adhesion junctions between epithelial cells due to forming complexes with nectin-3. Thus, CD155 effects on processes including migration, growth and cell motility [138].

Alternative splicing of *CD155* gene results in two transmembrane and two soluble forms of CD155 [137].

CD155 is recognized by a group of receptors expressed on T- and NK-cells: the activator receptor DNAX accessory molecule 1 (DNAM-1, or CD226) and inhibitory receptors T cell Ig and ITIM domain (TIGIT) and TACTILE (CD96) [139]. CD226 is an activating receptor that belongs to the Ig superfamily and is expressed on NK-cells, as well as T-cells, monocytes and B-cells. CD226 stimulates CD8+ T-cells and promotes NK-cell cytotoxicity and cytokine production [140]. TIGIT and CD96 receptors are expressed by T- and NK-cells after activation by chronic antigen exposure, for example, in malignant tumors [141]. These receptors compete with CD226 for binding to CD155 and inhibit T- and NK-cell functions in the TME [142]. TIGIT has been associated with NK-cell exhaustion in cancer patients. TIGIT blockade prevented NK-cell exhaustion and promoted NK-cell-dependent tumor immunity in several mouse models [143].

The human CD155 molecule was found to be significantly expressed in tumor cells of solid tumors [144]. In the absence of CD155 expression in tumor cells, there is a slower tumor growth and a decrease in metastasis, which indicates the importance of the role of CD155 in the tumor [145]. In GC cells, suppression of CD155 increased T-cell metabolism and IFN-γ production, while CD155 overexpression inhibited T-cell metabolism and IFN-γ production [146]. CD155 transcription may be induced by the activation of the Ras-Raf-MEK-ERK and the Sonic Hedgehog signaling pathways. CD155 overexpression leads to Ras mutated tumor cell proliferation due to G0/G1 phase shortening [137].

An association of increased CD155 expression with a poor prognosis has been noted in several malignant tumors [147,148,149,150]. In GC, an association of increased expression of CD155 with poor OS was observed [131,151].

There is evidence of an association of CD155 expression with clinical characteristics of the tumor. Thus, CD155 expression in some types of solid tumors was significantly associated with tumor size, depth of invasion, and TNM stage [147,148,152,153]. In GC, increased expression of sCD155 in blood serum was associated with the TNM stage [154].

Thus, in several cancers, increased expression of CD155 is associated with poor survival and other unfavorable clinical parameters. The available data indicate the perspectives of CD155 inhibition as a target for immune therapy [155,156].

### 2.9. Siglec-15

Sialic acid-binding immunoglobulin-type lectins (Siglecs) is a family of cell surface proteins with an essential role in the regulation of immune homeostasis. Siglecs recognizes a different kind of sialic acids, that are expressed on mammalian cells as a mechanism to distinguish self and non-self. Some pathogens can employ inhibitory Siglecs to attenuate the immune response and improve their survival. The dysregulation of Siglecs is associated with various diseases ranging from autoimmunity to infections and cancer [157]. Siglec-15 is a type I transmembrane protein consisting of two Ig-like domains, a transmembrane domain containing a lysine residue and a short cytoplasmic tail [158]. Siglec-15 is a ligand for an unknown inhibitory receptor on cytotoxic T-cells, in the same way as PD-L1 on cancer cells or tumor stroma engages IC molecule PD-1 on T-cells [159]. Siglec-15 is encoded by the *SIGLEC15* gene located on the long arm of chromosome 18 at locus 12.3 (18q12.3). Siglec-15 associates with the activating adaptor proteins DNAX activation protein (DAP)12 and DAP10 via its lysine residue in the transmembrane domain [158]. Siglec-15 binds to Sialyl-Tn, a short O-glycan with a sialic acid residue whose overexpression is associated with various types of epithelial cancers [157]. Siglec-15 is expressed on macrophages and DCs of spleen and lymph nodes [158]. Compared with normal tissues, Siglec-15 up-regulation was widely observed in tumors [160]. Moreover, Siglec-15 is overexpressed on cancer cells and tumor-infiltrating macrophages/myeloid cells in contrast to its low expression on macrophages in normal tissues. M-CSF may be a major inducer of Siglec-15 expression on macrophages. Due to the fact that M-CSF is inducible from various stromal and hematopoietic cells by early inflammatory mediators such as TNF-α and IL-1, Siglec-15 upregulation on macrophages by M-CSF may represent an initial negative regulation of macrophages/myeloid cells on the initiation of innate and adaptive immunity. Siglec-15 expression is mutually exclusive to PD-L1, partially due to its induction by M-CSF and downregulation by IFN-γ [161]. High Siglec-15 expression promotes hepatocellular carcinoma migration via CD44 interaction and osteosarcoma progression via the activation of the DUSP1/MAPK signaling pathway [162]. CD44 may be a cancer cell-associated ligand for Siglec-15, due to CD44 is overexpressed in various types of solid tumors [159].

High Siglec-15 mRNA expression correlated with favorable or unfavorable outcomes depending on the different type and subtype of cancer [160,163]. Siglec-15 overexpression was associated with poor OS in pancreatic ductal adenocarcinoma, kidney renal clear cell carcinoma and sarcoma [160]. In Li et al., high Siglec-15 expression was associated with poor OS in GC (HR = 4.87, 95% CI = 1.42–15.4, *p* = 0.006) [163]. At the same time, in other studies, the expression of Siglec-15 in GC was not associated with OS [160,164].

Quirino et al. reported the association of Siglec-15 expression with such clinicopathological characteristics as the TNM stage (*p* = 0.01), histological grade (*p* = 0.0022) and angiolymphatic invasion (*p* = 0.041) [164].

Siglec-15 may represent a novel pathway mediating cancer immune escape with the essential features, such as selective expression in the tumors, while almost absent in normal tissues; a strong tumor-induced immunosuppressive mechanism; and a targetable pathway that normalizes cancer immunity in the TME upon blockade [157]. Siglec-15 blockade can turn an immunosuppressive TME to an inflammatory site in some tumor models. [161]. Moreover, Siglec-15 represents an independent immune regulatory pathway from the PD-1/PD-L1 axis and shows a mutually exclusive expression profile with PD-L1 in human cancers, suggesting that it may provide a new therapeutic strategy for patients resistant to anti-PD therapy [157]. Altogether, these results suggest Siglec-15 as a potential candidate for cancer immunotherapy. An anti-Siglec-15 mAb was created, which, under the name NC318, is in ongoing phase 1 clinical trial (NCT03665285) [165].

### 2.10. ADAM17

ADAMs (a disintegrin and metalloproteinase) is a family of transmembrane and secreted proteins that play an essential role in the regulation of cell phenotype through effects on signaling, migration, cell adhesion and proteolysis [166]. ADAM17, a member of the ADAM family, also known as tumor necrosis factor-α converting enzyme (TACE), is a sheddase of various proteins including cytokines and cytokine receptors, growth factors, and adhesion molecules [167]. ADAM17 is protein of 824 amino acids, and its gene *ADAM17* is mapped on chromosome 2p25 [168]. ADAM17 controls the immune and inflammatory response through pro-TNF-α activation and is also important during development for the activation of membrane-associated epidermal growth factor receptor (EGFR) ligands [169]. ADAM17 mediates the activation of HER2, HER3, HER4 and EGFR receptors due to release of HER ligands including EGF, HB-EGF, heregulin, betacelluin and TGF-α [170]. ADAM17 influences on endothelial cell proliferation, invasion, network formation and activation of MMP-2, thereby regulating angiogenesis [171]. ADAM17 activates the TGF-β/Smad signaling pathway, which promotes EMT, as well as proliferation, migration, and invasion of GC cells [172]. Increased ADAM17 expression promotes GC progression through activation of Notch and/or Wnt signaling pathways [173].

The co-inhibitory receptor Tim-3 is a target of ADAM10 and ADAM17-mediated ectodomain shedding leading to a soluble form of Tim-3 [174]. It has also been shown that ADAM10 and ADAM17 cleave PD-L1 from the surface of tumor cells and extracellular vesicles. As a result of cleavage, an active fragment sPD-L1 is produced, which induces apoptosis in CD8+ T-cells and prevents tumor cells destruction by CD8+ T-cells. A decreased ratio of PD-L1 protein to mRNA in the tumor is associated with poor prognosis and correlates with increased expression of ADAM10 and ADAM17 in a variety of malignancies. This may explain the discrepancy between PD-L1 IHC and response to PD-1/PD-L1 inhibitors [175]. The expression level of mRNA and ADAM17 protein in gastric tissues is significantly higher than in normal gastric mucosa [176].

ADAM17 expression is associated with poor survival (HR = 2.04, 95% CI = 1.66–2.52, *p* < 0.001), lymph node metastasis (OR = 5.47, 95% CI = 3.98–7.51, *p* < 0.001) and distant metastasis (OR = 3.50, 95% CI = 1.79–6.87, *p* < 0.001) in many types of tumors [177]. In a meta-analysis by Ni et al. increased expression of ADAM17 in GC was significantly associated with poor survival, TNM stage and lymph node metastasis [178]. In a meta-analysis of the expression of ADAM10 and ADAM17 in GC, their increased expression was associated with T3-4 cancer more than T1-2 cancer (OR = 0.29, 95% CI = 0.21–0.40, *p* < 0.0001), N-positive cancer more than N-negative cancer (OR = 4.36, 95% CI = 2.25–8.45, *p* < 0.0001), cancer with distant metastasis more than cancer without metastasis (OR = 0.09, 95% CI = 0.02–0.37, *p* = 0.0008) [179]. Therefore, based on meta-analyses data, there is an association of increased ADAM17 expression in a number of tumor types, including GC, with the poor OS, TNM stage and lymph node metastasis.

ADAM17 inhibition leads to a clinically significant effect, however, due to frequently observed toxicity, the corresponding drugs have not yet entered the practice [180,181]. Some ADAM17 antibodies showing good results in model systems have not yet been tested in clinical trials [182]. It should be noted the recently developed new approach to inhibit ADAM17 in NK-cells using antibodies that enhance their anti-tumor activity [183].

Thus, the analysis of published data leads to the conclusion that the expression of the main part of the examined ICs (PD-L1, B7-H3, B7-H4, IDO1, CD155, and ADAM17) is associated with the prognosis of the development of solid tumors, including GC. Increased expression of these ICs is associated with worse patient survival and other unfavorable clinical parameters. At the same time, the expression of Gal-3 and -9 demonstrates a contradictory relationship with the prognosis of the development of solid tumors in different studies. There are insufficient data to conclude on the relationship between CEACAM1 and Siglec-15 expression and clinical characteristics.

On the other hand, inhibition of the most studied PD-L1, B7-H3, B7-H4 leads to clinically significant therapeutic results. The available data indicate that CD155 inhibition is promising as a target for immune therapy. The unsatisfactory results of clinical studies of several anti-IDO1 drugs were unexpected, which indicates a lack of understanding of the role of IDO1 in immune suppression and the need for further research in this direction. The situation is similar to the Galectins. In the light of one of the recent works [92], for Gal-9, a solution can be found that consists in inhibiting not only Gal-9. Similar results with a more detailed study can be obtained for other ICs. ADAM17 stands apart as it is not an IC, but it can influence their functioning. Although its inhibitors, suitable for use in clinical practice, have not yet been found, the presence of clinically significant effects inspires optimism.

## 3. Immune Checkpoints as Biomarkers of GC

The search for works with the expression of analyzed ICs in GC was carried out on the PubMed, PMC, Omicsonline, and Embase databases. For greater reliability of the data on the relationship between ICs expression and prognosis and the characteristics of such relationship, we tried to rely mainly on the results of meta-analyses. A detailed description of the considered works is given in Table 2.

### 3.1. PD-L1

PD-L1 expression is used in clinical practice as a marker for predicting the response to therapy with ICI directed primarily to the PD-1/PD-L1 axis, including among patients with GC [1,2]. At the same time, the assessment of PD-L1 expression can serve as a marker for prognosis of survival during therapy with inhibitors of the IC PD-1/PD-L1 in the second and more lines: PD-L1 expression was associated with better OS (HR = 0.82, 95% CI = 0.67–0.99, *p* = 0.04) [27].

The results of all analyzed meta-analyses of PD-L1 expression in GC showed an association of its expression with a poor prognosis of disease development, while the HR value ranged from 1.39 to 1.74 [17,18,19,20,21].

PD-L1 expression is also associated with the clinical and pathological characteristics of GC. The most significant meta-analysis results were obtained for the association with lymph node metastasis (OR = 2.61, 95% CI = 1.78–3.84), TNM stage (OR = 2.28, 95% CI = 1.39–3.74, *p* = 0.006) [21], tumor size (OR = 1.87, 95% CI = 1.25–2.78, *p* = 0.002) [19], and venous invasion (*p* = 0.0003) [18].

PD-L1 expression for prognostic purposes can be measured not only in the tumor, but also in the blood serum of patients with GC. In Shigemori et al. study PD-L1 expression was measured in both tumor tissues and serum by ELISA. Multivariate analyses showed that both elevated tissue PD-L1 and serum sPD-L1 were independent prognostic factors for poor OS; the obtained HR values turned out to be close: (HR = 4.28, 95% CI = 1.43–12.8, *p* = 0.0094) and (HR = 11.2, 95% CI = 3.44–36.7, *p* = 0.0001), respectively [25]. Ito et al. reported, that PD-L1 expression measured in preoperative peripheral blood by qRT-PCR was an independent poor prognostic factor for OS (HR = 1.81, 95% CI = 1.15–2.78, *p* < 0.05) [26].

The results considered lead to the conclusion about PD-L1 expression as a candidate for prognostic markers of GC with moderate-risk, an important property of which is the possibility of minimally invasive use.

### 3.2. B7-H3

There are still few studies on B7-H3 expression in GC. The available results indicate an association of increased B7-H3 expression in tumor tissue with low OS (*p* = 0.003), as well as with TNM stage (*p* = 0.000), depth of infiltration (*p* = 0.001), and lymph node metastasis (*p* = 0.020) [40]. An increased B7-H3 expression in peripheral blood was associated with low OS in GC (HR = 1.56, 95% CI = 1.01–2.54, *p* = 0.046) [41].

The available data indicate that further studies of B7-H3 among patients with GC are promising for elucidating the possibility of using its expression as a prognostic marker.

### 3.3. B7-H4

In all publications found, increased expression of B7-H4 was associated with a poor prognosis of GC (OR = 1.63, 95% CI = 1.30–2.03), as it is reflected in the meta-analysis [61]. The most significant results on the association of B7-H4 expression in tumors with a poor prognosis of GC were obtained in studies by Jiang et al. (RR = 1.85, 95% CI = 1.15–2.96, *p* = 0.0087) and Arigami et al. (HR = 1.49, 95% CI = 1.03–2.17, *p* = 0.035) [62,63]. Similar results were obtained when measuring B7-H4 expression level in the blood of patients with GC (HR = 2.01, 95% CI = 1.08–5.03, *p* = 0.024) and in the serum of such patients (HR = 1.925, 95% CI = 1.033–3.857, *p* = 0.039) [66,67].

B7-H4 expression is also associated with certain clinical and pathological tumor characteristics. Thus, Jiang et al. study shown association with myometrial invasion (*p* = 0.004), lymph node metastasis (*p* < 0.0001), recurrence (*p* = 0.003) [62]. Arigami et al. reported that B7-H4 expression in blood of GC patients significantly correlated with depth of infiltration (*p* = 0.006), lymph node metastasis (*p* = 0.001), TNM stage (*p* < 0.001), lymphatic invasion (*p* < 0.001), venous invasion (*p* = 0.010) [66].

Therefore, B7-H4 expression is a very promising candidate in markers with moderate risk (HR = 1.5–2) for survival prognosis of GC patients.

### 3.4. Galectin-3

Meta-analysis of Gal-3 expression in GC showed that decreased Gal-3 expression was significantly associated with a poor prognosis (HR = 0.49, 95% CI = 0.36–0.67, *p* < 0.001), lymphatic vessel invasion (OR = 0.48, 95% CI = 0.26–0.89, *p* = 0.018), the poor TNM stage (OR = 0.47, 95% CI = 0.32–0.40, *p* < 0.001), greater depth of invasion (OR = 0.33, 95% CI = 0.21–0.51, *p* < 0.001) and the poor degree of differentiation (OR = 0.10, 95% CI = 0.04–0.25, *p* < 0.001) [84]. Among the individual studies, it is worth noting Okada et al. study, in which decreased Gal-3 expression was associated with a poor prognosis also (RR = 3.831, 95% CI = 1.574–9.329, *p* = 0.0031), the degree of differentiation (*p* < 0.0001), lymph node metastasis (*p* = 0.0495), lymphatic invasion (*p* = 0.0086) and TNM stage (*p* = 0.0433) [71]. Kim et al. reported that Gal-3 expression was associated with better OS (*p* = 0.006), lower depth of invasion (*p* < 0.001), absence of lymph node metastasis (*p* = 0.001), absence of distant metastasis (*p* = 0.004), better TNM stage (*p* < 0.001) and absence of lymphovascular invasion (*p* = 0.035) [85]. However, it should be noted that the level of Gal-3 expression in serum correlated with lymph node metastasis (*p* = 0.001) and distant metastasis (*p* < 0.001) [88].

Although most of the available studies on GC indicate the association of decreased expression of Gal-3 with a poor prognosis; however, they are few and there are conflicting results, including among data on other types of tumors. The current situation requires further research to understand the prospects of using the expression of Gal-3 as a prognostic marker.

### 3.5. Galectin-9

Among the studies of Gal-9 expression in GC, the most impressive results were shown by Choi et al.; in their study Gal-9 expression was associated with better OS (HR = 0.51, 95% CI = 0.35–0.76, *p* = 0.001), and the absence of Gal-9 expression was associated with greater depth of infiltration (*p* < 0.001), lymph node metastasis (*p* < 0.001), and TNM stage (*p* < 0.001) [101]. It is also worth noting the study of Jiang et al., who showed an association of increased Gal-9 expression with better OS (*p* = 0.002); in addition, decreased expression was associated with lymph-vascular invasion (*p* = 0.034), lymph node metastasis (*p* = 0.009), distant metastasis (*p* = 0.002) and TNM stage (*p* = 0.043) [100].

The situation with the relationship between the expression of Gal-9 and the survival of patients with GC is similar to that observed in the study of Gal-3. Some studies find better survival with increased expression of Gal-9, and there are also conflicting results in different studies (discussed in Section 1). Further studies of Gal-9 expression are needed to characterize the association with the prognosis of solid tumors, including GC.

### 3.6. IDO1

As evidenced by the results presented in the previous section, increased expression of IDO1 in many tumor types, including GC, is associated with worse OS and clinical characteristics indicating a poor prognosis. From the point of view of considering the expression of IDO1 as a biomarker, it should be noted that Liu et al. showed an association of IDO1 expression with a poor prognosis of GC (HR = 1.596, 95% CI = 1.156–2.204, *p* = 0.005), depth of infiltration (*p* = 0.045) and lymph node metastasis (*p* < 0.001) [111]. Similar results on the association of IDO1 expression with a poor prognosis of GC were obtained by Nishi et al. (HR = 2.75, 95% CI = 1.01–7.58, *p* < 0.05) [113].

So far, few studies have been devoted to the study of IDO1 expression in connection with the prognosis of GC. Nevertheless, in combination with the results for other types of cancer, the data obtained indicate that further work is promising to establish the parameters of the relationship between IDO1 expression and the prognosis of GC development. The available data indicate a moderate risk of poor prognosis with overexpression of IDO1.

### 3.7. CEACAM1,CD155 and Siglec-15

Data on the expression of CEACAM1 and CD155 in relation to survival, including among patients with GC, are contradictory. Therefore, at the moment, it is premature to consider their expression as candidates for biomarkers. There are still little data on Siglec-15. Further research is needed.

### 3.8. ADAM17

In all analyzed studies with a significant association of ADAM17 expression with survival, ADAM17 expression was associated with a poor prognosis of GC. So, in a meta-analysis by Ni et al. ADAM17 expression was associated with poor OS (HR = 2.04, 95% CI = 1.66–2.50), TNM stage (OR = 4.09, 95% CI = 1.85–9.04) and lymph node metastasis (OR = 3.08, 95% CI = 1.13–8.36) [178]. Significant HR values were obtained by Zhang et al.: in their study ADAM17 expression was associated with poor OS (HR = 5.87, 95% CI = 1.59–20.52, *p* = 0.008), degree of tumor differentiation (*p* = 0.006), depth of invasion (*p* < 0.0001), lymph node metastasis (*p* = 0.02), distant metastasis (*p* = 0.02) and TNM stage (*p* = 0.03) [185]. In Shou et al., HR values for the association of increased ADAM17 expression with poor OS were lower (HR = 2.067, 95% CI = 1.475–2.883, *p* = 0.000); however, high levels of significance of the relationship between expression and other clinical parameters of poor prognosis were observed: tumor size (*p* = 0.000), depth of invasion (*p* = 0.000), TNM stage (*p* = 0.000), lymph node metastasis (*p* = 0.000) and distant metastasis (*p* = 0.000) [184]. Li et al. obtained similar results: ADAM17 expression was associated with poor OS (HR = 2.239, 95% CI = 1.516–3.305, *p* < 0.001) and lymph node metastasis (OR = 2.161, 95% CI = 1.115–4.190, *p* = 0.022) [173]. Hence, an association of increased ADAM17 expression with reduced survival in GC patients has been identified, possibly with a higher than moderate risk. At the same time, the parameters of association with other clinical indicators of poor prognosis, such as metastasis, remain to be elucidated.

So, the considered members of the B7 family—B7-H1 (PD-L1), B7-H3, B7-H4, as well as IDO1 and ADAM17, can be recognized as candidates for prognosis markers for the survival of patients with GC. The risk of poor survival associated with their increased expression is moderate, except for ADAM17, which may be associated with a higher risk. The expression of the remaining analyzed ICs—Gal-3 and -9, CEACAM1, CD155 and Siglec-15—requires further in-depth study.

## 4. Conclusions

The published data on the relationship between the expression of several ICs—PD-L1, B7-H3, B7-H4, IDO1, Gal-3 and -9, CEACAM1, CD155, Siglec-15, and the ADAM17 immune response modulator—with the prognosis of cancer tumors, including GC, are analyzed. These data are compared with the results of clinical studies on their inhibition. Expression of PD-L1, B7-H3, B7-H4, IDO1, CD155, and ADAM17 is associated with the prognosis of tumors, including GC. Increased expression of the most studied ICs—PD-L1, B7-H3, and B7-H4—is associated with poor patient survival and other unfavorable clinical parameters, and their inhibition leads to clinically significant therapeutic results. The available data indicate that CD155 inhibition is promising as a target for immune therapy. The unsatisfactory results of clinical studies of several anti-IDO1 drugs turned out to be unexpected, in light of the confident relationship between IDO1 expression and clinical parameters, which indicates an insufficient understanding of the role of IDO1 in immune suppression and the need for further research in this direction. Expression of Gal-3 and -9, as well as CEACAM1 and Siglec-15, demonstrates a contradictory relationship with the development of solid tumors in different studies. The lack of satisfactory results from clinical studies of inhibitors of these ICs additionally indicates the complex nature of their functioning. Thus, according to the data of Yang et al., the resulting effect of Gal-9 may depend on the ratios of the three immune components, and in order to achieve a positive effect, the accumulation of Treg cells in the tumor space due to Gal-9 inhibition should be eliminated [92]. Accordingly, when studying the relationship between the expression of Gal-9 and the survival rate of patients with malignant tumors, one should, apparently, analyze the expression of other participants in the immune response to the tumor. This also applies to other ICs that do not show a reproducible relationship with clinical indicators. In general, on the combination of an IC expression association with clinical features and a response to their therapeutic inhibition, three IC groups can be distinguished. In the first group, the association with clinical features corresponds to the clinically significant therapeutic results (PD-L1, B7-H3, B7-H4). In the second group, the controversial association of expression with the development of tumors corresponds to unsatisfactory results of clinical studies of inhibitors of these ICs (Gal-3 and -9, CEACAM1). The third unexpected combination is a confident association of IDO1 expression with clinical features and unsatisfactory results of clinical studies. This case is the most mysterious for interpretation. It can be assumed that, in the IDO1 inhibition, a side reaction occurs similar to the accumulation of Treg cells in the TME under the inhibition of Gal-9, but that there are no multicomponent interactions, apparently inherent for the second group. In the first group, if such complications occur, it is not often.

Based on the available data, the considered members of the B7 family—B7-H1 (PD-L1), B7-H3, B7-H4, as well as IDO1 and ADAM17—can be recognized as candidates for prognosis markers for the survival of patients with GC.

## Figures and Tables

**Table 1:** Immune checkpoint names and their receptors or substrates.

Molecule	Alternative Name	Receptor/Substrate
B7-H1	CD274/PD-L1/PDCD1L1	PD-1
B7-H3	CD276/B7RP-2	TLT-2 (?)
B7-H4	VTCN1/B7S1/B7x	BTLA (?)
Galectin-3	MAC-2	LAG3/LGALS3BP
Galectin-9	Ecalectin	Tim-3/PD-1/CD44
IDO1	INDO	* Tryptophan
CEACAM1	CD66a/BGP	Tim-3
CD155	PVR/Necl-5	TIGIT/CD96/CD226
Siglec-15	CD33L3/HsT1361	CD44/Sialyl-Tn
ADAM17	CD156b/TACE	* Pro-TNF-a/Pro-TGF- a/Notch1/Pro-Amphiregulin/Pro-HB-EGF/Pro-epiregulin/Neuregulin 1/IL6 Receptor/etc.

Footnotes: (?)—putative receptor; *—substrate.

**Table 2:** Immune checkpoints as potential GC biomarkers.

Checkpoint	Characteristics of Checkpoints as Candidates for GC Markers	Materials/ Method	Reference
B7-H1	Increased expression is associated with poor 3-year OS (HR = 1.23, 95% CI = 1.02–1.49, *p* = 0.028), poor 5-year OS (HR = 1.39, 95% CI = 1.14–1.69, *p* = 0.001), lymph node metastasis (OR = 1.73, 95% CI = 1.18–2.54, *p* < 0.01)	Meta-analysis GC tissue/IHC	[17]
	Increased expression is associated with poor OS (HR = 1.46, 95% CI = 1.08–1.98, *p* = 0.01), greater depth of infiltration (*p* = 0.03), lymph node metastasis (*p* = 0.03), venous invasion (*p* = 0.0003)	Meta-analysis GC tissue/IHC	[18]
	Increased expression is associated with poor OS (HR = 1.64, 95% CI = 1.11–2.43, *p* = 0.01), large tumor size (OR = 1.87, 95% CI = 1.25–2.78, *p* = 0.002), lymph node metastasis (OR = 2.17, 95% CI = 1.04–4.52, *p* = 0.04).	Meta-analysis GC tissue/IHC	[19]
	Increased expression is associated with poor OS (HR = 1.60, 95% CI = 1.09–2.36, *p* = 0.012)	Meta-analysis GC tissue/IHC	[20]
	Increased expression is associated with poor OS (HR = 1.74, 95% CI = 1.40–2.17, *p* = 0.146), lymph node metastasis (OR = 2.61, 95% CI = 1.78–3.84, *p* = 0.004), higher TNM stage (OR = 2.28, 95% CI = 1.39–3.74, *p* = 0.006)	Meta-analysis GC tissue, Serum/ IHC, ELISA	[21]
	Better OS in patients with PD-L1-positive tumors treated with ICIs (HR = 0.82, 95% CI = 0.67–0.99, *p* = 0.04)	Meta-analysis GC tissue/IHC	[27]
	Increased expression is associated with better OS (HR = 0.753, 95% CI = 0.584–0.971, *p* = 0.029), less advanced depth of infiltration (*p* = 0.001), absence of distant metastasis (*p* = 0.029), lower TNM stage (*p* = 0.01)	GC tissue/IHC	[13]
	Increased expression is associated with poor OS (HR = 1.81, 95% CI = 1.15–2.78, *p* < 0.05)	Blood/ qRT-PCR	[26]
	Increased expression in GC tissue is associated with poor OS (HR = 4.28, 95% CI = 1.43–12.8, *p* = 0.0094), depth of infiltration (*p* = 0.0003), vessel involvement (*p* < 0.0001), lymphatic vessel involvement (*p* = 0.0005), lymph node metastasis (*p* = 0.023), peritoneal metastasis (*p* = 0.0098) Increased expression in serum is associated with poor OS (HR = 11.2, 95% CI = 3.44–36.7, *p* = 0.0001)	GC tissue, Serum/ IHC, ELISA	[25]
B7-H3	Increased expression is associated with poor OS (*p* = 0.003), higher TNM stage (*p* = 0.000), greater depth of infiltration (*p* = 0.001), lymph node metastasis (*p* = 0.020)	GC tissue/IHC	[40]
	Increased expression is associated with better OS (RR = 2.803, 95% CI = 1.051–7.477, *p* = 0.040)	GC tissue/IHC	[42]
	Increased expression is associated with greater depth of infiltration (*p* = 0.005)	GC tissue/IHC	[34]
	Increased stromal expression is associated with greater depth of infiltration (*p* = 0.013)	GC tissue/IHC	[35]
	Increased expression is associated with poor OS (HR = 1.56, 95% CI = 1.01–2.54, *p* = 0.046), higher TNM stage (*p* = 0.013)	Blood/qRT-PCR	[41]
B7-H4	Increased expression is associated with poor prognosis (OR = 1.63, 95% CI = 1.30–2.03)	Meta-analysis GC tissue, blood, serum/IHC, qRT-PCR, ELISA	[61]
	Increased expression is associated with poor prognosis (RR = 1.85, 95% CI = 1.15–2.96, *p* = 0.0087), myometrial invasion (*p* = 0.004), lymph node metastasis (*p* < 0.0001), recurrence (*p* = 0.003)	GC tissue/IHC	[62]
	Increased expression is associated with poor prognosis (HR = 1.49, 95% CI = 1.03–2.17, *p* = 0.035), higher TNM stage (*p* = 0.04)	GC tissue/IHC	[63]
	Increased expression is associated with poor prognosis (HR = 1.41, 95% CI = 1.01–1.98, *p* = 0.049), lymph node metastasis (*p* = 0.007), higher TNM stage (*p* = 0.004), greater depth of infiltration (*p* = 0.011)	GC tissue/IHC	[64]
	Decreased expression is associated with better OS in NACT (neoadjuvant chemotherapy) group (*p* = 0.031)	GC tissue/IHC	[65]
	Increased expression is associated with poor prognosis (HR = 2.01, 95% CI = 1.08–5.03, *p* = 0.024), greater depth of infiltration (*p* = 0.006), lymph node metastasis (*p* = 0.001), higher TNM stage (*p* < 0.001), lymphatic invasion (*p* < 0.001), venous invasion (*p* = 0.010)	Blood/RT-PCR	[66]
	Increased expression is associated with poor prognosis (HR = 1.925, 95% CI = 1.033–3.857, *p* = 0.039), large tumor size (*p* = 0.002), lymph node metastasis (*p* = 0.001), greater depth of infiltration (*p* = 0.041) higher TNM stage (*p* < 0.001)	Serum/ ELISA	[67]
Galectin-3	Decreased expression is associated with poor prognosis (HR = 0.49, 95% CI = 0.36–0.67, *p* < 0.001), lymphatic vessel invasion (OR = 0.48, 95% CI = 0.26–0.89, *p* = 0.018), higher TNM stage (OR = 0.47, 95% CI = 0.32–0.40, *p* < 0.001), greater depth of infiltration (OR = 0.33, 95% CI = 0.21–0.51, *p* < 0.001), poorer differentiation grade (OR = 0.10, 95% CI = 0.04–0.25, *p* < 0.001)	Meta-analysis GC tissue, serum/IHC, ELISA	[84]
	Increased expression is associated with better OS (*p* = 0.006), less advanced depth of infiltration (*p* < 0.001), absence of lymph node metastasis (*p* = 0.001), absence of distant metastasis (*p* = 0.004), lower TNM stage (*p* < 0.001), absence of lymphovascular invasion (*p* = 0.035)	GC tissue/IHC	[85]
	Decreased expression is associated with poor prognosis (RR = 3.831, 95% CI = 1.574–9.329, *p* = 0.0031), diffuse type (*p* < 0.0001), poor tumor grade (*p* < 0.0001), lymph node metastasis (*p* = 0.0495), lymphatic invasion (*p* = 0.0086), higher TNM stage (*p* = 0.0433)	GC tissue/IHC	[71]
	Increased expression is associated with higher TNM stage (*p* = 0.038), poor differentiation (*p* = 0.001), lymph node metastasis (*p* = 0.022)	GC tissue/RT-PCR	[86]
	Increased expression is associated with higher TNM stage (*p* = 0.0019)	GC tissue/IHC	[87]
	Increased expression is associated with lymph node metastasis (*p* = 0.001), distant metastasis (*p* < 0.001)	Serum/ ELISA	[88]
Galectin-9	Increased expression is associated with better OS (HR = 0.51, 95% CI = 0.35–0.76, *p* = 0.001) Gal-9 negativity is associated with greater depth of infiltration (*p* < 0.001), lymph node metastasis (*p* < 0.001), higher TNM stage (*p* < 0.001)	GC tissue/IHC	[101]
	Increased expression is associated with poor OS (*p* = 0.0028), lymph node metastasis (*p* = 0.0060), higher TNM stage (*p* = 0.0292), blood vessel invasion (*p* = 0.0410)	GC tissue/IHC	[94]
	Increased expression is associated with better OS (*p* = 0.002) Decreased expression is associated with lymph-vascular invasion (*p* = 0.034), lymph node metastasis (*p* = 0.009), distant metastasis (*p* = 0.002), higher TNM staging (*p* = 0.043)	GC tissue/IHC	[100]
IDO1	Increased expression is associated with poor OS (HR = 1.596, 95% CI = 1.156–2.204, *p* = 0.005), greater depth of infiltration (*p* = 0.045), lymph node metastasis (*p* < 0.001)	GC tissue/IHC	[111]
	Increased expression is associated with poor OS (*p* = 0.0059)	GC tissue/IHC	[112]
	Increased expression is associated with poor OS (HR = 2.75, 95% CI = 1.01–7.58, *p* < 0.05)	GC tissue/IHC	[113]
	Increased expression is associated with greater depth of infiltration (*p* = 0.016), lymph node metastasis (*p* = 0.038)	GC tissue/IHC	[117]
	Increased expression is associated with poor OS (*p* = 0.043), large tumor size (*p* = 0.044), greater depth of infiltration (*p* = 0.027)	GC tissue/IHC	[114]
	Increased expression is associated with greater depth of infiltration (*p* = 0.016), lymph node metastasis (*p* = 0.046)	GC tissue/IHC	[118]
	Increased expression is associated with well/moderately differentiated histology (*p* < 0.001), intestinal type (*p* < 0.001), absence of vascular invasion (*p* = 0.012), lower TNM stage (*p* = 0.007)	GC tissue/IHC	[116]
	Increased expression is associated with lower rate of metastasis (*p* = 0.032), lower rate of recurrence (*p* = 0.010)	GC tissue/IHC	[115]
CEACAM1	Increased expression is associated with poor OS (*p* = 0.001)	GC tissue/IHC	[131]
	Loss of CEACAM1 is associated with poor OS (HR = 3.472, 95% CI = 1.508–8.00, *p* = 0.03), peritoneal dissemination after gastrectomy (HR = 3.711, 95% CI = 1.253–10.995, *p* = 0.018)	GC tissue/IHC	[132]
	Increased expression is associated with lymph node metastasis (*p* < 0.05), higher TNM stage (*p* < 0.05)	GC tissue/IHC	[133]
	Increased expression is associated with lymph node metastasis (*p* < 0.05)	GC tissue/IHC	[134]
CD155	Increased expression is associated with poor OS (*p* = 0.001)	GC tissue/IHC	[131]
	Increased TIGIT and CD155 expression were associated with poor OS (*p* = 0.011)	KM Plotter GC database	[151]
	Increased expression is associated with higher TNM stage (*p* < 0.05)	Serum/ ELISA	[154]
Siglec-15	Increased expression is associated with poor OS (HR = 4.87, 95% CI = 1.42–15.4, *p* = 0.006)	TCGA and GEO databases	[163]
	Increased expression is associated with TNM stage (*p* = 0.01), histological grade (*p* = 0.0022), angiolymphatic invasion (*p* = 0.041)	GC tissue/IHC	[164]
ADAM17	Increased expression is associated with poor OS (HR = 2.04, 95% CI = 1.66–2.50, *p* = 0.299), higher TNM stage (OR = 4.09, 95% CI = 1.85–9.04, *p* = 0.000), lymph node metastasis (OR = 3.08, 95% CI = 1.13–8.36, *p* = 0.007)	Meta-analysis GC tissue/IHC	[178]
	Increased ADAM10 and ADAM17 expression were associated with greater depth of infiltration (OR = 0.29, 95% CI = 0.21 to 0.40, *p* < 0.0001), lymph node metastasis (OR = 4.36, 95% CI = 2.25 to 8.45, *p* < 0.0001), distant metastasis (OR = 0.09, 95% CI = 0.02 to 0.37, *p* = 0.0008)	Meta-analysis GC tissue/IHC	[179]
	Increased expression is associated with poor prognosis (HR = 2.067, 95% CI = 1.475–2.883, *p* = 0.000), large tumor size (*p* = 0.000), greater depth of invasion (*p* = 0.000), higher TNM stage (*p* = 0.000), diffuse type (*p* = 0.000), vessel invasion (*p* = 0.000), lymph node metastasis (*p* = 0.000), distant metastasis (*p* = 0.000)	GC tissue/IHC	[184]
	Increased expression is associated with poor prognosis (HR = 5.87, 95% CI = 1.59–20.52, *p* = 0.008), poor differentiation (*p* = 0.006), greater depth of invasion (*p* < 0.0001), lymph node metastasis (*p* = 0.02), distant metastasis (*p* = 0.02), higher TNM stage (*p* = 0.03)	GC tissue/IHC	[185]
	Increased expression is associated with poor OS (*p* = 0.019), poor DFS (HR = 1.61, 95% CI = 0.93–2.79, *p* = 0.038), large tumor size (*p* = 0.04), lymph node metastasis (*p* = 0.003), vascular invasion (*p* = 0.015), recurrence (*p* = 0.032)	GC tissue/IHC	[186]
	Increased expression is associated with poor prognosis (HR = 2.239, 95% CI = 1.516–3.305, *p* < 0.001) (AUC = 0.618, *p* = 0.006), lymph node metastasis (OR = 2.161, 95% CI = 1.115–4.190, *p* = 0.022)	GC tissue/IHC	[173]
	Increased expression is associated with greater depth of invasion (*p* = 0.007), distant metastasis (*p* = 0.047), higher TNM stage (*p* = 0.001)	GC tissue/IHC	[187]
	Increased expression is associated with poor prognosis (*p* = 0.007), lymph node metastasis (*p* = 0.005), higher TNM stage (*p* = 0.002)	GC tissue/IHC	[188]

Footnotes: IHC—immunohistochemistry; ELISA—the enzyme-linked immunosorbent assay; RT-PCR—real-time PCR; GC—gastric cancer; TNM—classification of malignant tumors; HR—hazard ratio; OS—overall survival; AUC—area under curve; OR—odds ratio; CI—confidence interval.

## Data Availability

The cited works can be found on the PubMed, PMC, Omicsonline, and Embase databases.

## References

[1] Doroshow D.B., Bhalla S., Beasley M.B., Sholl L.M., Kerr K.M., Gnjatic S., Wistuba I.I., Rimm D.L., Tsao M.S., Hirsch F.R. (2021). PD-L1 as a biomarker of response to immune-checkpoint inhibitors. Nat. Rev. Clin. Oncol..

[2] Patel S.P., Kurzrock R. (2015). PD-L1 Expression as a Predictive Biomarker in Cancer Immunotherapy. Mol. Cancer Ther..

[3] Bolandi N., Derakhshani A., Hemmat N., Baghbanzadeh A., Asadzadeh Z., Afrashteh Nour M., Brunetti O., Bernardini R., Silvestris N., Baradaran B. (2021). The Positive and Negative Immunoregulatory Role of B7 Family: Promising Novel Targets in Gastric Cancer Treatment. Int. J. Mol. Sci..

[4] Cao Y., Wang X., Jin T., Tian Y., Dai C., Widarma C., Song R., Xu F. (2020). Immune checkpoint molecules in natural killer cells as potential targets for cancer immunotherapy. Signal Transduct. Target. Ther..

[5] Zhang Y., Zheng J. (2020). Functions of Immune Checkpoint Molecules Beyond Immune Evasion. Adv. Exp. Med. Biol..

[6] Qin S., Xu L., Yi M., Yu S., Wu K., Luo S. (2019). Novel immune checkpoint targets: Moving beyond PD-1 and CTLA-4. Mol. Cancer.

[7] Sung H., Ferlay J., Siegel R.L., Laversanne M., Soerjomataram I., Jemal A., Bray F. (2021). Global Cancer Statistics 2020: GLOBOCAN Estimates of Incidence and Mortality Worldwide for 36 Cancers in 185 Countries. CA. Cancer J. Clin..

[8] Etemadi A., Safiri S., Sepanlou S.G., Ikuta K., Bisignano C., Shakeri R., Amani M., Fitzmaurice C., Nixon M., Abbasi N. (2020). The global, regional, and national burden of stomach cancer in 195 countries, 1990–2017: A systematic analysis for the Global Burden of Disease study 2017. Lancet Gastroenterol. Hepatol..

[9] Keir M.E., Butte M.J., Freeman G.J., Sharpe A.H. (2008). PD-1 and Its Ligands in Tolerance and Immunity. Annu. Rev. Immunol..

[10] Liu J., Chen Z., Li Y., Zhao W., Wu J.B., Zhang Z. (2021). PD-1/PD-L1 Checkpoint Inhibitors in Tumor Immunotherapy. Front. Pharmacol..

[11] Francisco L.M., Sage P.T., Sharpe A.H. (2010). The PD-1 pathway in tolerance and autoimmunity. Immunol. Rev..

[12] Hirano F., Kaneko K., Tamura H., Dong H., Wang S., Ichikawa M., Rietz C., Flies D.B., Lau J.S., Zhu G. (2005). Blockade of B7-H1 and PD-1 by monoclonal antibodies potentiates cancer therapeutic immunity. Cancer Res..

[13] Böger C., Behrens H.-M., Mathiak M., Krüger S., Kalthoff H., Röcken C. (2016). PD-L1 is an independent prognostic predictor in gastric cancer of Western patients. Oncotarget.

[14] Wu P., Wu D., Li L., Chai Y., Huang J. (2015). PD-L1 and Survival in Solid Tumors: A Meta-Analysis. PLoS ONE.

[15] Xiang X., Yu P.-C., Long D., Liao X.-L., Zhang S., You X.-M., Zhong J.-H., Li L.-Q. (2018). Prognostic value of PD-L1 expression in patients with primary solid tumors. Oncotarget.

[16] Dai C., Wang M., Lu J., Dai Z., Lin S., Yang P., Tian T., Liu X., Min W., Dai Z. (2017). Prognostic and predictive values of PD-L1 expression in patients with digestive system cancer: A meta-analysis. Onco. Targets. Ther..

[17] Qiu Z., Du Y. (2021). Clinicopathological and prognostic significance of programmed death ligant-1 expression in gastric cancer: A meta-analysis. J. Gastrointest. Oncol..

[18] Gu L., Chen M., Guo D., Zhu H., Zhang W., Pan J., Zhong X., Li X., Qian H., Wang X. (2017). PD-L1 and gastric cancer prognosis: A systematic review and meta-analysis. PLoS ONE.

[19] Zhang M., Dong Y., Liu H., Wang Y., Zhao S., Xuan Q., Wang Y., Zhang Q. (2016). The clinicopathological and prognostic significance of PD-L1 expression in gastric cancer: A meta-analysis of 10 studies with 1,901 patients. Sci. Rep..

[20] Feng X.-S., Wang X.-S., Wang Y.-F., Hu X.-C., Yan J.-Q., Wang W., Yang R.-J., Feng Y.-Y., Gao S.-G., Liu Y.-X. (2016). Prognostic significance of PD-L1 expression in patients with gastric cancer in East Asia: A meta-analysis. Onco. Targets. Ther..

[21] Xu F., Feng G., Zhao H., Liu F., Xu L., Wang Q., An G. (2015). Clinicopathologic Significance and Prognostic Value of B7 Homolog 1 in Gastric Cancer: A Systematic Review and Meta-Analysis. Medicine.

[22] Kim D.H., Bae G.E., Suh K.S., Ryuman D., Song K.S., Kim J.S., Lee S.-I., Yeo M.-K. (2020). Clinical Significance of Tumor and Immune Cell PD-L1 Expression in Gastric Adenocarcinoma. In Vivo.

[23] Kawazoe A., Kuwata T., Kuboki Y., Shitara K., Nagatsuma A.K., Aizawa M., Yoshino T., Doi T., Ohtsu A., Ochiai A. (2017). Clinicopathological features of programmed death ligand 1 expression with tumor-infiltrating lymphocyte, mismatch repair, and Epstein-Barr virus status in a large cohort of gastric cancer patients. Gastric Cancer.

[24] Huang P., Hu W., Zhu Y., Wu Y., Lin H. (2021). The Prognostic Value of Circulating Soluble Programmed Death Ligand-1 in Cancers: A Meta-Analysis. Front. Oncol..

[25] Shigemori T., Toiyama Y., Okugawa Y., Yamamoto A., Yin C., Narumi A., Ichikawa T., Ide S., Shimura T., Fujikawa H. (2019). Soluble PD-L1 Expression in Circulation as a Predictive Marker for Recurrence and Prognosis in Gastric Cancer: Direct Comparison of the Clinical Burden Between Tissue and Serum PD-L1 Expression. Ann. Surg. Oncol..

[26] Ito S., Fukagawa T., Noda M., Hu Q., Nambara S., Shimizu D., Kuroda Y., Eguchi H., Masuda T., Sato T. (2018). Prognostic Impact of Immune-Related Gene Expression in Preoperative Peripheral Blood from Gastric Cancer Patients. Ann. Surg. Oncol..

[27] Roviello G., Corona S.P., D’Angelo A., Rosellini P., Nobili S., Mini E. (2020). Immune Checkpoint Inhibitors in Pre-Treated Gastric Cancer Patients: Results from a Literature-Based Meta-Analysis. Int. J. Mol. Sci..

[28] Chapoval A.I., Ni J., Lau J.S., Wilcox R.A., Flies D.B., Liu D., Dong H., Sica G.L., Zhu G., Tamada K. (2001). B7-H3: A costimulatory molecule for T cell activation and IFN-gamma production. Nat. Immunol..

[29] Hashiguchi M., Kobori H., Ritprajak P., Kamimura Y., Kozono H., Azuma M. (2008). Triggering receptor expressed on myeloid cell-like transcript 2 (TLT-2) is a counter-receptor for B7-H3 and enhances T cell responses. Proc. Natl. Acad. Sci. USA.

[30] Suh W.-K., Gajewska B.U., Okada H., Gronski M.A., Bertram E.M., Dawicki W., Duncan G.S., Bukczynski J., Plyte S., Elia A. (2003). The B7 family member B7-H3 preferentially down-regulates T helper type 1–mediated immune responses. Nat. Immunol..

[31] Prasad D.V.R., Nguyen T., Li Z., Yang Y., Duong J., Wang Y., Dong C. (2004). Murine B7-H3 Is a Negative Regulator of T Cells. J. Immunol..

[32] Tekle C., Nygren M.K., Chen Y.-W., Dybsjord I., Nesland J.M., Maelandsmo G.M., Fodstad O. (2012). B7-H3 contributes to the metastatic capacity of melanoma cells by modulation of known metastasis-associated genes. Int. J. cancer.

[33] Dai W., Shen G., Qiu J., Zhao X., Gao Q. (2014). Aberrant expression of B7-H3 in gastric adenocarcinoma promotes cancer cell metastasis. Oncol. Rep..

[34] Li Y., Yang X., Wu Y., Zhao K., Ye Z., Zhu J., Xu X., Zhao X., Xing C. (2017). B7-H3 promotes gastric cancer cell migration and invasion. Oncotarget.

[35] Ulase D., Behrens H.-M., Krüger S., Zeissig S., Röcken C. (2021). Gastric Carcinomas with Stromal B7-H3 Expression Have Lower Intratumoural CD8+ T Cell Density. Int. J. Mol. Sci..

[36] Li Y., Yang X., Yao P., Shen W., Wu Y., Ye Z., Zhao K., Chen H., Cao J., Xing C. (2019). B7-H3 increases the radioresistance of gastric cancer cells through regulating baseline levels of cell autophagy. Am. J. Transl. Res..

[37] Ye Z., Zheng Z., Li X., Zhu Y., Zhong Z., Peng L., Wu Y. (2016). B7-H3 Overexpression Predicts Poor Survival of Cancer Patients: A Meta-Analysis. Cell. Physiol. Biochem..

[38] Li G., Quan Y., Che F., Wang L. (2018). B7-H3 in tumors: Friend or foe for tumor immunity?. Cancer Chemother. Pharmacol..

[39] Zhang X., Fang C., Zhang G., Jiang F., Wang L., Hou J. (2017). Prognostic value of B7-H3 expression in patients with solid tumors: A meta-analysis. Oncotarget.

[40] Zhan S., Liu Z., Zhang M., Guo T., Quan Q., Huang L., Guo L., Cao L., Zhang X. (2020). Overexpression of B7-H3 in α-SMA-Positive Fibroblasts Is Associated with Cancer Progression and Survival in Gastric Adenocarcinomas. Front. Oncol..

[41] Arigami T., Uenosono Y., Hirata M., Yanagita S., Ishigami S., Natsugoe S. (2011). B7-H3 expression in gastric cancer: A novel molecular blood marker for detecting circulating tumor cells. Cancer Sci..

[42] Wu C.-P., Jiang J.-T., Tan M., Zhu Y.-B., Ji M., Xu K.-F., Zhao J.-M., Zhang G.-B., Zhang X.-G. (2006). Relationship between co-stimulatory molecule B7-H3 expression and gastric carcinoma histology and prognosis. World J. Gastroenterol..

[43] Loo D., Alderson R.F., Chen F.Z., Huang L., Zhang W., Gorlatov S., Burke S., Ciccarone V., Li H., Yang Y. (2012). Development of an Fc-enhanced anti-B7-H3 monoclonal antibody with potent antitumor activity. Clin. Cancer Res..

[44] Safety Study of MGA271 in Refractory Cancer. https://clinicaltrials.gov/ct2/show/NCT01391143.

[45] Safety Study of Enoblituzumab (MGA271) in Combination with Pembrolizumab or MGA012 in Refractory Cancer. https://clinicaltrials.gov/ct2/show/NCT02475213.

[46] Enoblituzumab Plus Retifanlimab or Tebotelimab in Head and Neck Cancer. https://clinicaltrials.gov/ct2/show/NCT04634825.

[47] Aggarwal C., Joshua A., Ferris R., Antonia S., Rahma O., Tolcher A., Cohen R.B., Lou Y., Hauke R., Vogelzang N. (2018). A phase 1, open-label, dose-escalation study of enoblituzumab in combination with pembrolizumab in patients with select solid tumors. J. Immunother. Cancer.

[48] Shenderov E., Demarzo A., Boudadi K., Allaf M., Wang H., Chapman C., Pavlovich C., Bivalacqua T., O’Neal T.S., Harb R. (2018). Phase II neoadjuvant and immunologic study of B7-H3 targeting with enoblituzumab in localized intermediate- and high-risk prostate cancer. J. Clin. Oncol..

[49] Ohaegbulam K.C., Liu W., Jeon H., Almo S.C., Zang X. (2017). Tumor-expressed immune checkpoint B7x promotes cancer progression and antigen-specific CD8 T cell exhaustion and suppressive innate immune cells. Oncotarget.

[50] Podojil J.R., Miller S.D. (2017). Potential targeting of B7-H4 for the treatment of cancer. Immunol. Rev..

[51] Choi I.-H., Zhu G., Sica G.L., Strome S.E., Cheville J.C., Lau J.S., Zhu Y., Flies D.B., Tamada K., Chen L. (2003). Genomic Organization and Expression Analysis of B7-H4, an Immune Inhibitory Molecule of the B7 Family. J. Immunol..

[52] Watanabe N., Gavrieli M., Sedy J.R., Yang J., Fallarino F., Loftin S.K., Hurchla M.A., Zimmerman N., Sim J., Zang X. (2003). BTLA is a lymphocyte inhibitory receptor with similarities to CTLA-4 and PD-1. Nat. Immunol..

[53] Zang X., Loke P., Kim J., Murphy K., Waitz R., Allison J.P. (2003). B7x: A widely expressed B7 family member that inhibits T cell activation. Proc. Natl. Acad. Sci. USA.

[54] Wang J.Y., Wang W.P. (2020). B7-H4, a promising target for immunotherapy. Cell. Immunol..

[55] Sica G.L., Choi I.H., Zhu G., Tamada K., Wang S.D., Tamura H., Chapoval A.I., Flies D.B., Bajorath J., Chen L. (2003). B7-H4, a molecule of the B7 family, negatively regulates T cell immunity. Immunity.

[56] Ni L., Dong C. (2017). New B7 Family Checkpoints in Human Cancers. Mol. Cancer Ther..

[57] Wu H., Wang X., Mo N., Zhang L., Yuan X., Lü Z. (2018). B7-Homolog 4 Promotes Epithelial-Mesenchymal Transition and Invasion of Bladder Cancer Cells via Activation of Nuclear Factor-κB. Oncol. Res. Featur. Preclin. Clin. Cancer Ther..

[58] Chen C., Zhu W.-D., Xie F., Huang J.-A. (2016). Nuclear localization of B7-H4 in pulmonary adenocarcinomas presenting as a solitary pulmonary nodule. Oncotarget.

[59] Shan Z., Yan Z., Peng L., Cheng P., Teng Y., Mao F., Fan K., Zhuang Y., Zhao Y. (2021). Granulocyte-Macrophage Colony-Stimulating Factor-Activated Neutrophils Express B7-H4 That Correlates with Gastric Cancer Progression and Poor Patient Survival. J. Immunol. Res..

[60] Song X., Shao Y., Gu W., Xu C., Mao H., Pei H., Jiang J. (2016). Prognostic role of high B7-H4 expression in patients with solid tumors: A meta-analysis. Oncotarget.

[61] Cui Y., Li Z. (2016). B7-H4 is Predictive of Poor Prognosis in Patients with Gastric Cancer. Med. Sci. Monit..

[62] Jiang J., Zhu Y., Wu C., Shen Y., Wei W., Chen L., Zheng X., Sun J., Lu B., Zhang X. (2010). Tumor expression of B7-H4 predicts poor survival of patients suffering from gastric cancer. Cancer Immunol. Immunother..

[63] Arigami T., Uenosono Y., Ishigami S., Hagihara T., Haraguchi N., Natsugoe S. (2011). Clinical significance of the B7-H4 coregulatory molecule as a novel prognostic marker in gastric cancer. World J. Surg..

[64] Geng Y., Wang H., Lu C., Li Q., Xu B., Jiang J., Wu C. (2015). Expression of costimulatory molecules B7-H1, B7-H4 and Foxp3+ Tregs in gastric cancer and its clinical significance. Int. J. Clin. Oncol..

[65] Maskey N., Li K., Hu M., Xu Z., Peng C., Yu F., Cao H., Chen J., Li Y., Yang G. (2014). Impact of neoadjuvant chemotherapy on lymphocytes and co-inhibitory B7-H4 molecule in gastric cancer: Low B7-H4 expression associates with favorable prognosis. Tumor Biol..

[66] Arigami T., Uenosono Y., Hirata M., Hagihara T., Yanagita S., Ishigami S., Natsugoe S. (2010). Expression of B7-H4 in blood of patients with gastric cancer predicts tumor progression and prognosis. J. Surg. Oncol..

[67] Shi H., Ji M., Wu J., Zhou Q., Li X., Li Z., Zheng X., Xu B., Zhao W., Wu C. (2014). Serum B7-H4 expression is a significant prognostic indicator for patients with gastric cancer. World J. Surg. Oncol..

[68] Sachdev J.C., Bauer T.M., Chawla S.P., Pant S., Patnaik A., Wainberg Z.A., Inamdar S.P., Marina N., Sun S., Schmidt M. (2019). Phase 1a/1b study of first-in-class B7-H4 antibody, FPA150, as monotherapy in patients with advanced solid tumors. J. Clin. Oncol..

[69] Yang R.-Y., Rabinovich G.A., Liu F.-T. (2008). Galectins: Structure, function and therapeutic potential. Expert Rev. Mol. Med..

[70] Sciacchitano S., Lavra L., Morgante A., Ulivieri A., Magi F., De Francesco G., Bellotti C., Salehi L., Ricci A. (2018). Galectin-3: One Molecule for an Alphabet of Diseases, from A to Z. Int. J. Mol. Sci..

[71] Okada K., Shimura T., Suehiro T., Mochiki E., Kuwano H. (2006). Reduced galectin-3 expression is an indicator of unfavorable prognosis in gastric cancer. Anticancer Res..

[72] Dong R., Zhang M., Hu Q., Zheng S., Soh A., Zheng Y., Yuan H. (2017). Galectin-3 as a novel biomarker for disease diagnosis and a target for therapy (Review). Int. J. Mol. Med..

[73] Newlaczyl A.U., Yu L.-G. (2011). Galectin-3—A jack-of-all-trades in cancer. Cancer Lett..

[74] de Oliveira J.T., de Matos A.J., Gomes J., Vilanova M., Hespanhol V., Manninen A., Rutteman G., Chammas R., Gärtner F., Bernardes E.S. (2010). Coordinated expression of galectin-3 and galectin-3-binding sites in malignant mammary tumors: Implications for tumor metastasis. Glycobiology.

[75] Chocarro L., Blanco E., Zuazo M., Arasanz H., Bocanegra A., Fernández-Rubio L., Morente P., Fernández-Hinojal G., Echaide M., Garnica M. (2021). Understanding LAG-3 Signaling. Int. J. Mol. Sci..

[76] Farhad M., Rolig A.S., Redmond W.L. (2018). The role of Galectin-3 in modulating tumor growth and immunosuppression within the tumor microenvironment. Oncoimmunology.

[77] Compagno D., Tiraboschi C., Garcia J.D., Rondón Y., Corapi E., Velazquez C., Laderach D.J. (2020). Galectins as Checkpoints of the Immune System in Cancers, Their Clinical Relevance, and Implication in Clinical Trials. Biomolecules.

[78] Nakahara S., Raz A. (2007). Regulation of cancer-related gene expression by galectin-3 and the molecular mechanism of its nuclear import pathway. Cancer Metastasis Rev..

[79] Elad-Sfadia G., Haklai R., Balan E., Kloog Y. (2004). Galectin-3 Augments K-Ras Activation and Triggers a Ras Signal That Attenuates ERK but Not Phosphoinositide 3-Kinase Activity. J. Biol. Chem..

[80] Thijssen V.L., Heusschen R., Caers J., Griffioen A.W. (2015). Galectin expression in cancer diagnosis and prognosis: A systematic review. Biochim. Biophys. Acta-Rev. Cancer.

[81] Wang Y., Liu S., Tian Y., Wang Y., Zhang Q., Zhou X., Meng X., Song N. (2018). Prognostic role of galectin-3 expression in patients with solid tumors: A meta-analysis of 36 eligible studies. Cancer Cell Int..

[82] Wang C., Zhou X., Ma L., Zhuang Y., Wei Y., Zhang L., Jin S., Liang W., Shen X., Li C. (2019). Galectin-3 may serve as a marker for poor prognosis in colorectal cancer: A meta-analysis. Pathol.-Res. Pract..

[83] Shao Q., He J., Chen Z., Wu C. (2020). Prognostic role of galectins expression in patients with hepatic cancer. Medicine.

[84] Long B., Yu Z., Zhou H., Ma Z., Ren Y., Zhan H., Li L., Cao H., Jiao Z. (2018). Clinical characteristics and prognostic significance of galectins for patients with gastric cancer: A meta-analysis. Int. J. Surg..

[85] Kim S.J., Kim D.C., Kim M.C., Jung G.J., Kim K.H., Jang J.S., Kwon H.C., Kim Y.M., Jeong J.S. (2012). Fascin expression is related to poor survival in gastric cancer. Pathol. Int..

[86] Dong W.-G., Yu Q.-F., Xu Y., Fan L.-F. (2008). Li-cadherin is Inversely Correlated with Galectin-3 Expression in Gastric Cancer. Dig. Dis. Sci..

[87] Miyazaki J., Hokari R., Kato S., Tsuzuki Y., Kawaguchi A., Nagao S., Itoh K., Miura S. (2002). Increased expression of galectin-3 in primary gastric cancer and the metastatic lymph nodes. Oncol. Rep..

[88] Li Y. (2015). Serum Galectin-3 as a Potential Marker for Gastric Cancer. Med. Sci. Monit..

[89] Chou F.-C., Chen H.-Y., Kuo C.-C., Sytwu H.-K. (2018). Role of Galectins in Tumors and in Clinical Immunotherapy. Int. J. Mol. Sci..

[90] Heusschen R., Griffioen A.W., Thijssen V.L. (2013). Galectin-9 in tumor biology: A jack of multiple trades. Biochim. Biophys. Acta-Rev. Cancer.

[91] Moar P., Tandon R. (2021). Galectin-9 as a biomarker of disease severity. Cell. Immunol..

[92] Yang R., Sun L., Li C.-F., Wang Y.-H., Yao J., Li H., Yan M., Chang W.-C., Hsu J.-M., Cha J.-H. (2021). Galectin-9 interacts with PD-1 and TIM-3 to regulate T cell death and is a target for cancer immunotherapy. Nat. Commun..

[93] Oomizu S., Arikawa T., Niki T., Kadowaki T., Ueno M., Nishi N., Yamauchi A., Hattori T., Masaki T., Hirashima M. (2012). Cell surface galectin-9 expressing Th cells regulate Th17 and Foxp3+ Treg development by galectin-9 secretion. PLoS ONE.

[94] Wang Y., Zhao E., Zhang Z., Zhao G., Cao H. (2018). Association between Tim-3 and Gal-9 expression and gastric cancer prognosis. Oncol. Rep..

[95] Lee B.-H., Park Y., Kim J.-H., Kang K.-W., Lee S.-J., Kim S.J., Kim B.S. (2021). Prognostic Value of Galectin-9 Relates to Programmed Death-Ligand 1 in Patients with Multiple Myeloma. Front. Oncol..

[96] Liu Y., Liu Z., Fu Q., Wang Z., Fu H., Liu W., Wang Y., Xu J. (2017). Galectin-9 as a prognostic and predictive biomarker in bladder urothelial carcinoma. Urol. Oncol. Semin. Orig. Investig..

[97] Chen P., He Y., Zhou C. (2021). P47.13 Galectin-9, A Novel Prognostic Factor in Small Cell Lung Cancer. J. Thorac. Oncol..

[98] Zhou X., Sun L., Jing D., Xu G., Zhang J., Lin L., Zhao J., Yao Z., Lin H. (2018). Galectin-9 Expression Predicts Favorable Clinical Outcome in Solid Tumors: A Systematic Review and Meta-Analysis. Front. Physiol..

[99] Wang K., Chen Z., Wu R., Yin J., Fan M., Xu X. (2018). Prognostic Role of High Gal-9 Expression in Solid Tumours: A Meta-Analysis. Cell. Physiol. Biochem..

[100] Jiang J., Jin M.-S., Kong F., Cao D., Ma H.-X., Jia Z., Wang Y.-P., Suo J., Cao X. (2013). Decreased galectin-9 and increased Tim-3 expression are related to poor prognosis in gastric cancer. PLoS ONE.

[101] Choi S.I., Seo K.W., Kook M.C., Kim C.G., Kim Y.W., Cho S.J. (2017). Prognostic value of tumoral expression of galectin-9 in gastric cancer. Turkish J. Gastroenterol..

[102] Schulz H., Kuhn C., Hofmann S., Mayr D., Mahner S., Jeschke U., Schmoeckel E. (2018). Overall survival of ovarian cancer patients is determined by expression of galectins-8 and -9. Int. J. Mol. Sci..

[103] Ye Z., Yue L., Shi J., Shao M., Wu T. (2019). Role of IDO and TDO in Cancers and Related Diseases and the Therapeutic Implications. J. Cancer.

[104] Zhai L., Ladomersky E., Lenzen A., Nguyen B., Patel R., Lauing K.L., Wu M., Wainwright D.A. (2018). IDO1 in cancer: A Gemini of immune checkpoints. Cell. Mol. Immunol..

[105] Yu C.-P., Fu S.-F., Chen X., Ye J., Ye Y., Kong L.-D., Zhu Z. (2018). The Clinicopathological and Prognostic Significance of IDO1 Expression in Human Solid Tumors: Evidence from a Systematic Review and Meta-Analysis. Cell. Physiol. Biochem..

[106] Prendergast G.C., Malachowski W.J., Mondal A., Scherle P., Muller A.J. (2018). Indoleamine 2,3-Dioxygenase and Its Therapeutic Inhibition in Cancer. Int. Rev. Cell Mol. Biol..

[107] Opitz C.A., Somarribas Patterson L.F., Mohapatra S.R., Dewi D.L., Sadik A., Platten M., Trump S. (2020). The therapeutic potential of targeting tryptophan catabolism in cancer. Br. J. Cancer.

[108] Liu M., Wang X., Wang L., Ma X., Gong Z., Zhang S., Li Y. (2018). Targeting the IDO1 pathway in cancer: From bench to bedside. J. Hematol. Oncol..

[109] Prendergast G.C., Mondal A., Dey S., Laury-Kleintop L.D., Muller A.J. (2018). Inflammatory Reprogramming with IDO1 Inhibitors: Turning Immunologically Unresponsive ‘Cold’ Tumors ‘Hot’. Trends in Cancer.

[110] Wang S., Wu J., Shen H., Wang J. (2020). The prognostic value of IDO expression in solid tumors: A systematic review and meta-analysis. BMC Cancer.

[111] Liu H., Shen Z., Wang Z., Wang X., Zhang H., Qin J., Qin X., Xu J., Sun Y. (2016). Increased expression of IDO associates with poor postoperative clinical outcome of patients with gastric adenocarcinoma. Sci. Rep..

[112] Lu S., Wang L.J., Lombardo K., Kwak Y., Kim W.H., Resnick M.B. (2020). Expression of Indoleamine 2, 3-dioxygenase 1 (IDO1) and Tryptophanyl-tRNA Synthetase (WARS) in Gastric Cancer Molecular Subtypes. Appl. Immunohistochem. Mol. Morphol..

[113] Nishi M., Yoshikawa K., Higashijima J., Tokunaga T., Kashihara H., Takasu C., Ishikawa D., Wada Y., Shimada M. (2018). The Impact of Indoleamine 2,3-dioxygenase (IDO) Expression on Stage III Gastric Cancer. Anticancer Res..

[114] Li F., Sun Y., Huang J., Xu W., Liu J., Yuan Z. (2019). CD4/CD8 + T cells, DC subsets, Foxp3, and IDO expression are predictive indictors of gastric cancer prognosis. Cancer Med..

[115] Patil P.A., Blakely A.M., Lombardo K.A., Machan J.T., Miner T.J., Wang L.-J., Marwaha A.S., Matoso A. (2018). Expression of PD-L1, indoleamine 2,3-dioxygenase and the immune microenvironment in gastric adenocarcinoma. Histopathology.

[116] Kim J.W., Nam K.H., Ahn S.-H., Park D.J., Kim H.-H., Kim S.H., Chang H., Lee J.-O., Kim Y.J., Lee H.S. (2016). Prognostic implications of immunosuppressive protein expression in tumors as well as immune cell infiltration within the tumor microenvironment in gastric cancer. Gastric Cancer.

[117] Zhang R., Liu H., Li F., Li H., Yu J., Ren X. (2013). The Correlation Between the Subsets of Tumor Infiltrating Memory T Cells and the Expression of Indoleamine 2,3-Dioxygenase in Gastric Cancer. Dig. Dis. Sci..

[118] Li F., Huang J., Li S., Li H., Yu J., Ren X., Liu J. (2014). The subsets of dendritic cells and memory T cells correspond to indoleamine 2,3-dioxygenase in stomach tumor microenvironment. Tumor Biol..

[119] Meireson A., Devos M., Brochez L. (2020). IDO Expression in Cancer: Different Compartment, Different Functionality?. Front. Immunol..

[120] Schalper K.A., Carvajal-Hausdorf D., McLaughlin J., Altan M., Velcheti V., Gaule P., Sanmamed M.F., Chen L., Herbst R.S., Rimm D.L. (2017). Differential Expression and Significance of PD-L1, IDO-1, and B7-H4 in Human Lung Cancer. Clin. Cancer Res..

[121] Öztürk S., Kalter V., Roessner P.M., Sunbul M., Seiffert M. (2021). IDO1-Targeted Therapy Does Not Control Disease Development in the Eµ-TCL1 Mouse Model of Chronic Lymphocytic Leukemia. Cancers.

[122] Beauchemin N., Draber P., Dveksler G., Gold P., Gray-Owen S., Grunert F., Hammarstrom S., Holmes K.V., Karlsson A., Kuroki M. (1999). Redefined Nomenclature for Members of the Carcinoembryonic Antigen Family. Exp. Cell Res..

[123] Dankner M., Gray-Owen S.D., Huang Y.-H., Blumberg R.S., Beauchemin N. (2017). CEACAM1 as a Multi-Purpose Target for Cancer Immunotherapy. Oncoimmunology.

[124] Han Z.-W., Lyv Z.-W., Cui B., Wang Y.-Y., Cheng J.-T., Zhang Y., Cai W.-Q., Zhou Y., Ma Z.-W., Wang X.-W. (2020). The old CEACAMs find their new role in tumor immunotherapy. Investig. New Drugs.

[125] Huang Y.H., Zhu C., Kondo Y., Anderson A.C., Gandhi A., Russell A., Dougan S.K., Petersen B.S., Melum E., Pertel T. (2015). CEACAM1 regulates TIM-3-mediated tolerance and exhaustion. Nature.

[126] Calinescu A., Turcu G., Nedelcu R.I., Brinzea A., Hodorogea A., Antohe M., Diaconu C., Bleotu C., Pirici D., Jilaveanu L.B. (2018). On the Dual Role of Carcinoembryonic Antigen-Related Cell Adhesion Molecule 1 (CEACAM1) in Human Malignancies. J. Immunol. Res..

[127] Ergün S., Kilik N., Ziegeler G., Hansen A., Nollau P., Götze J., Wurmbach J.H., Horst A., Weil J., Fernando M. (2000). CEA-related cell adhesion molecule 1: A potent angiogenic factor and a major effector of vascular endothelial growth factor. Mol. Cell.

[128] Yang F., Zeng Z., Li J., Ren X., Wei F. (2021). TIM-3 and CEACAM1 are Prognostic Factors in Head and Neck Squamous Cell Carcinoma. Front. Mol. Biosci..

[129] Thies A., Moll I., Berger J., Wagener C., Brümmer J., Schulze H.-J., Brunner G., Schumacher U. (2002). CEACAM1 expression in cutaneous malignant melanoma predicts the development of metastatic disease. J. Clin. Oncol..

[130] Thöm I., Schult-Kronefeld O., Burkholder I., Schuch G., Andritzky B., Kastendieck H., Edler L., Wagener C., Bokemeyer C., Schumacher U. (2009). Expression of CEACAM-1 in pulmonary adenocarcinomas and their metastases. Anticancer Res..

[131] Wang J.-B., Li P., Liu X.-L., Zheng Q.-L., Ma Y.-B., Zhao Y.-J., Xie J.-W., Lin J.-X., Lu J., Chen Q.-Y. (2020). An immune checkpoint score system for prognostic evaluation and adjuvant chemotherapy selection in gastric cancer. Nat. Commun..

[132] Takeuchi A., Yokoyama S., Nakamori M., Nakamura M., Ojima T., Yamaguchi S., Mitani Y., Shively J.E., Yamaue H. (2019). Loss of CEACAM1 is associated with poor prognosis and peritoneal dissemination of patients with gastric cancer. Sci. Rep..

[133] Shi J.-F., Xu S.-X., He P., Xi Z.-H. (2014). Expression of carcinoembryonic antigen-related cell adhesion molecule 1(CEACAM1) and its correlation with angiogenesis in gastric cancer. Pathol.-Res. Pract..

[134] Zhou C.-J., Liu B., Zhu K.-X., Zhang Q.-H., Zhang T.-G., Xu W.-H., Wang H.-B., Yu W.-H., Qu Y.-D., Wang H.-J. (2009). The different expression of carcinoembryonic antigen-related cell adhesion molecule 1 (CEACAM1) and possible roles in gastric carcinomas. Pathol.-Res. Pract..

[135] Lee E.H., Lee J., Hur M., Park H.-Y., Yum H.I., Nam H., Oh M.-Y., Choi H., Kim J., Cho B.C. (2019). MG1124, a novel CEACAM1-targeted monoclonal antibody, has therapeutic potential as a combination partner of PD-1 inhibitors in NSCLC patients. Ann. Oncol..

[136] Cho H.J., Lee J.-C., Park H.-Y., Yang W.S., Nam H.-M., Ryu J.H., Oh Y., Hur M. (2020). 1064P Efficacy of a novel anti-CEACAM1 monoclonal antibody and CEACAM1 up-regulation in tumour-infiltrating lymphocytes (TILs) of cancer patients. Ann. Oncol..

[137] Molfetta R., Zitti B., Lecce M., Milito N.D., Stabile H., Fionda C., Cippitelli M., Gismondi A., Santoni A., Paolini R. (2020). CD155: A Multi-Functional Molecule in Tumor Progression. Int. J. Mol. Sci..

[138] Maier M.K., Seth S., Czeloth N., Qiu Q., Ravens I., Kremmer E., Ebel M., Müller W., Pabst O., Förster R. (2007). The adhesion receptor CD155 determines the magnitude of humoral immune responses against orally ingested antigens. Eur. J. Immunol..

[139] Chan C.J., Andrews D.M., Smyth M.J. (2012). Receptors that interact with nectin and nectin-like proteins in the immunosurveillance and immunotherapy of cancer. Curr. Opin. Immunol..

[140] de Andrade L.F., Smyth M.J., Martinet L. (2014). DNAM-1 control of natural killer cells functions through nectin and nectin-like proteins. Immunol. Cell Biol..

[141] O’Donnell J.S., Madore J., Li X.-Y., Smyth M.J. (2020). Tumor intrinsic and extrinsic immune functions of CD155. Semin. Cancer Biol..

[142] Deuss F.A., Watson G.M., Fu Z., Rossjohn J., Berry R. (2019). Structural Basis for CD96 Immune Receptor Recognition of Nectin-like Protein-5, CD155. Structure.

[143] Zhang Q., Bi J., Zheng X., Chen Y., Wang H., Wu W., Wang Z., Wu Q., Peng H., Wei H. (2018). Blockade of the checkpoint receptor TIGIT prevents NK cell exhaustion and elicits potent anti-tumor immunity. Nat. Immunol..

[144] Chandramohan V., Bryant J.D., Piao H., Keir S.T., Lipp E.S., Lefaivre M., Perkinson K., Bigner D.D., Gromeier M., McLendon R.E. (2017). Validation of an Immunohistochemistry Assay for Detection of CD155, the Poliovirus Receptor, in Malignant Gliomas. Arch. Pathol. Lab. Med..

[145] Li X.-Y., Das I., Lepletier A., Addala V., Bald T., Stannard K., Barkauskas D., Liu J., Aguilera A.R., Takeda K. (2018). CD155 loss enhances tumor suppression via combined host and tumor-intrinsic mechanisms. J. Clin. Investig..

[146] He W., Zhang H., Han F., Chen X., Lin R., Wang W., Qiu H., Zhuang Z., Liao Q., Zhang W. (2017). CD155T/TIGIT Signaling Regulates CD8+ T-cell Metabolism and Promotes Tumor Progression in Human Gastric Cancer. Cancer Res..

[147] Zhao K., Ma L., Feng L., Huang Z., Meng X., Yu J. (2021). CD155 Overexpression Correlates with Poor Prognosis in Primary Small Cell Carcinoma of the Esophagus. Front. Mol. Biosci..

[148] Luo C., Ye W., Hu J., Othmane B., Li H., Chen J., Zu X. (2021). A Poliovirus Receptor (CD155)-Related Risk Signature Predicts the Prognosis of Bladder Cancer. Front. Oncol..

[149] Zhang H., Yang Z., Du G., Cao L., Tan B. (2021). CD155-Prognostic and Immunotherapeutic Implications Based on Multiple Analyses of Databases Across 33 Human Cancers. Technol. Cancer Res. Treat..

[150] Li Y.-C., Zhou Q., Song Q.-K., Wang R.-B., Lyu S., Guan X., Zhao Y.-J., Wu J.-P. (2020). Overexpression of an Immune Checkpoint (CD155) in Breast Cancer Associated with Prognostic Significance and Exhausted Tumor-Infiltrating Lymphocytes: A Cohort Study. J. Immunol. Res..

[151] Xu D., Zhao E., Zhu C., Zhao W., Wang C., Zhang Z., Zhao G. (2020). TIGIT and PD-1 may serve as potential prognostic biomarkers for gastric cancer. Immunobiology.

[152] Yong H., Cheng R., Li X., Gao G., Jiang X., Cheng H., Zhou X., Zhao W. (2019). CD155 expression and its prognostic value in postoperative patients with breast cancer. Biomed. Pharmacother..

[153] Huang D.-W., Huang M., Lin X.-S., Huang Q. (2017). CD155 expression and its correlation with clinicopathologic characteristics, angiogenesis, and prognosis in human cholangiocarcinoma. Onco. Targets. Ther..

[154] Iguchi-Manaka A., Okumura G., Kojima H., Cho Y., Hirochika R., Bando H., Sato T., Yoshikawa H., Hara H., Shibuya A. (2016). Increased Soluble CD155 in the Serum of Cancer Patients. PLoS ONE.

[155] Ma W., Ma J., Lei T., Zhao M., Zhang M. (2019). Targeting immunotherapy for bladder cancer by using anti-CD3 × CD155 bispecific antibody. J. Cancer.

[156] Wu L., Mao L., Liu J.-F., Chen L., Yu G.-T., Yang L.-L., Wu H., Bu L.-L., Kulkarni A.B., Zhang W.-F. (2019). Blockade of TIGIT/CD155 Signaling Reverses T-cell Exhaustion and Enhances Antitumor Capability in Head and Neck Squamous Cell Carcinoma. Cancer Immunol. Res..

[157] Sun J., Lu Q., Sanmamed M.F., Wang J. (2021). Siglec-15 as an Emerging Target for Next-generation Cancer Immunotherapy. Clin. Cancer Res..

[158] Angata T., Tabuchi Y., Nakamura K., Nakamura M. (2007). Siglec-15: An immune system Siglec conserved throughout vertebrate evolution. Glycobiology.

[159] Angata T. (2020). Siglec-15: A potential regulator of osteoporosis, cancer, and infectious diseases. J. Biomed. Sci..

[160] Li Q., Huang Z., Chen Y., Yao H., Ke Z., He X., Qiu M., Wang M., Xiong Z., Yang S. (2020). Integrative Analysis of Siglec-15 mRNA in Human Cancers Based on Data Mining. J. Cancer.

[161] Wang J., Sun J., Liu L.N., Flies D.B., Nie X., Toki M., Zhang J., Song C., Zarr M., Zhou X. (2019). Siglec-15 as an immune suppressor and potential target for normalization cancer immunotherapy. Nat. Med..

[162] Lim J., Sari-Ak D., Bagga T. (2021). Siglecs as Therapeutic Targets in Cancer. Biology.

[163] Li B., Zhang B., Wang X., Zeng Z., Huang Z., Zhang L., Wei F., Ren X., Yang L. (2020). Expression signature, prognosis value, and immune characteristics of Siglec-15 identified by pan-cancer analysis. Oncoimmunology.

[164] Quirino M.W.L., Pereira M.C., de Souza M.D.F.D., da Rocha Pitta I., da Silva Filho A.F., de Souza Albuquerque M.S., de Barros Albuquerque A.P., Martins M.R., da Rocha Pitta M.G., de Melo Rêgo M.J.B. (2021). Immunopositivity for Siglec-15 in gastric cancer and its association with clinical and pathological parameters. Eur. J. Histochem..

[165] A Safety and Tolerability Study of NC318 in Subjects with Advanced or Metastatic Solid Tumors. https://clinicaltrials.gov/ct2/show/NCT03665285.

[166] Edwards D.R., Handsley M.M., Pennington C.J. (2008). The ADAM metalloproteinases. Mol. Aspects Med..

[167] Hassemer E.L., Endres B., Toonen J.A., Ronchetti A., Dubielzig R., Sidjanin D.J. (2013). ADAM17 transactivates EGFR signaling during embryonic eyelid closure. Invest. Ophthalmol. Vis. Sci..

[168] Gooz M. (2010). ADAM-17: The enzyme that does it all. Crit. Rev. Biochem. Mol. Biol..

[169] Blobel C.P. (2005). ADAMs: Key components in EGFR signalling and development. Nat. Rev. Mol. Cell Biol..

[170] Shen H., Li L., Zhou S., Yu D., Yang S., Chen X., Wang D., Zhong S., Zhao J., Tang J. (2016). The role of ADAM17 in tumorigenesis and progression of breast cancer. Tumor Biol..

[171] Göoz P., Göoz M., Baldys A., Hoffman S. (2009). ADAM-17 regulates endothelial cell morphology, proliferation, and in vitro angiogenesis. Biochem. Biophys. Res. Commun..

[172] Xu M., Zhou H., Zhang C., He J., Wei H., Zhou M., Lu Y., Sun Y., Ding J.W., Zeng J. (2016). ADAM17 promotes epithelial-mesenchymal transition via TGF-α/Smad pathway in gastric carcinoma cells. Int. J. Oncol..

[173] Li W., Wang D., Sun X., Zhang Y., Wang L., Suo J. (2019). ADAM17 promotes lymph node metastasis in gastric cancer via activation of the Notch and Wnt signaling pathways. Int. J. Mol. Med..

[174] Möller-Hackbarth K., Dewitz C., Schweigert O., Trad A., Garbers C., Rose-John S., Scheller J. (2013). A Disintegrin and Metalloprotease (ADAM) 10 and ADAM17 Are Major Sheddases of T Cell Immunoglobulin and Mucin Domain 3 (Tim-3). J. Biol. Chem..

[175] Orme J.J., Jazieh K.A., Xie T., Harrington S., Liu X., Ball M., Madden B., Charlesworth M.C., Azam T.U., Lucien F. (2020). ADAM10 and ADAM17 cleave PD-L1 to mediate PD-(L)1 inhibitor resistance. Oncoimmunology.

[176] Yoshimura T., Tomita T., Dixon M.F., Axon A.T.R., Robinson P.A., Crabtree J.E. (2002). ADAMs (a disintegrin and metalloproteinase) messenger RNA expression in Helicobacter pylori-infected, normal, and neoplastic gastric mucosa. J. Infect. Dis..

[177] Peng X., Hao B., Cao N., Wang J., Lv X., Zhang X. (2018). ADAM17 overexpression is associated with poorer clinical outcomes in cancer patients: A systematic review and meta-analysis. Oncotarget.

[178] Ni P., Yu M., Zhang R., He M., Wang H., Chen S., Duan G. (2020). Prognostic Significance of ADAM17 for Gastric Cancer Survival: A Meta-Analysis. Medicina.

[179] Sun X., Wang Y., Ma R., Li W. (2017). Clinical significance of ADAM10 and ADAM17 in gastric and colorectal cancers: A systematic review and meta-analysis. Int. J. Clin. Exp. Med..

[180] Moss M.L., Minond D. (2017). Recent Advances in ADAM17 Research: A Promising Target for Cancer and Inflammation. Mediators Inflamm..

[181] Saad M.I., Rose-John S., Jenkins B.J. (2019). Jenkins ADAM17: An Emerging Therapeutic Target for Lung Cancer. Cancers.

[182] Ye J., Yuen S.M., Murphy G., Xie R., Kwok H.F. (2017). Anti-tumor effects of a ‘human & mouse cross-reactive’ anti-ADAM17 antibody in a pancreatic cancer model in vivo. Eur. J. Pharm. Sci..

[183] Mishra H.K., Dixon K.J., Pore N., Felices M., Miller J.S., Walcheck B. (2021). Activation of ADAM17 by IL-15 Limits Human NK Cell Proliferation. Front. Immunol..

[184] Shou Z.-X., Jin X., Zhao Z.-S. (2012). Upregulated Expression of ADAM17 Is a Prognostic Marker for Patients with Gastric Cancer. Ann. Surg..

[185] Zhang T., Zhu W., Huang M., Fan R., Chen X. (2012). Prognostic value of ADAM17 in human gastric cancer. Med. Oncol..

[186] Aydin D., Bilici A., Yavuzer D., Kefeli U., Tan A., Ercelep O., Mert A., Yuksel S., Ozcelik M., Isik D. (2015). Prognostic significance of ADAM17 expression in patients with gastric cancer who underwent curative gastrectomy. Clin. Transl. Oncol..

[187] Sun J., Jiang J., Lu K., Chen Q., Tao D., Chen Z. (2017). Therapeutic potential of ADAM17 modulation in gastric cancer through regulation of the EGFR and TNF-α signalling pathways. Mol. Cell. Biochem..

[188] Fang W., Qian J., Wu Q., Chen Y., Yu G. (2017). ADAM-17 expression is enhanced by FoxM1 and is a poor prognostic sign in gastric carcinoma. J. Surg. Res..

